# Adverse effects, perceptions and attitudes related to BNT162b2, mRNA-1273 or JNJ-78436735 SARS-CoV-2 vaccines: Population-based cohort

**DOI:** 10.1038/s41541-023-00657-3

**Published:** 2023-04-24

**Authors:** Oliver Bürzle, Dominik Menges, Julian D. Maier, Daniel Schams, Milo A. Puhan, Jan Fehr, Tala Ballouz, Anja Frei

**Affiliations:** Epidemiology, Biostatistics and Prevention Institute (EBPI), University of Zurich (UZH), Zurich, Switzerland

**Keywords:** Viral infection, Drug safety, Epidemiology

## Abstract

Long-term control of SARS-CoV-2 requires effective vaccination strategies. This has been challenged by public mistrust and the spread of misinformation regarding vaccine safety. Better understanding and communication of the longer-term and comparative experiences of individuals in the general population following vaccination are required. In this population-based longitudinal study, we included 575 adults, randomly selected from all individuals presenting to a Swiss reference vaccination center, for receipt of BNT162b2, mRNA1273, or JNJ-78436735. We assessed the prevalence, onset, duration, and severity of self-reported adverse effects over 12 weeks following vaccination. We additionally evaluated participants’ perceptions of vaccines, trust in public health authorities and pharmaceutical companies, and compliance with public health measures. Most participants reported at least one adverse effect within 12 weeks following vaccination. Adverse effects were mostly mild or moderate, resolved within three days, and rarely resulted in anaphylaxis or hospitalizations. Female sex, younger age, higher education, and receipt of mRNA-1273 were associated with reporting adverse effects. Compared to JNJ-78436735 recipients, a higher proportion of mRNA vaccine recipients agreed that vaccination is important, and trusted public health authorities. Our findings provide real-world estimates of the prevalence of adverse effects following SARS-CoV-2 vaccination and highlight the importance of transparent communication to ensure the success of current or future vaccination campaigns.

## Introduction

Beginning with the first vaccinations against SARS-CoV-2 in December 2020, the largest global vaccination campaign in recent history captured public and professional attention for months. Apart from the obvious focus on efficacy, concerns about vaccine-related adverse effects dominated the professional and public discourse during the campaign. The fast-track authorization of the technologically new mRNA vaccines BNT162b2 (Pfizer-BioNTech) and mRNA-1273 (Moderna) in some countries and misinformation contributed to vaccine skepticism and hesitancy^1–5^. This highlights the importance of understanding and accurately communicating information regarding adverse effects, including vaccine safety profiles, to improve vaccine confidence and uptake.

The current body of evidence on adverse effects following SARS-CoV-2 vaccination consists mostly of data reported in randomized clinical trials (RCTs) and reports to government-based surveillance systems, such as the European EudraVigilance, US VAERS (Vaccine Adverse Event Reporting System), or Swiss ElViS (Electronic Vigilance System). Adverse effects reported in RCTs have been primarily mild and self-limited, with systemic reactions (e.g. fatigue, headache, pain) and local injection site reactions (e.g. pain, erythema, swelling) being the most frequently seen^6–8^. In contrast, severe adverse effects accounted for a significantly higher proportion of reports in governmental surveillance systems^9–11^. This was to be expected as reporting to surveillance systems is subject to several biases including underreporting of mild and common adverse effects and increased reporting of those which are severe or widely reported in the media^12,13^. Although RCTs provided important evidence on the safety of individual vaccines, they offered little side-by-side comparisons. Furthermore, RCTs yield data collected on selected populations raising the issue of how well this data correlates to “real-world” experiences. The few studies eliciting patient-reported symptoms following different vaccines in real-world settings often had cross-sectional designs and were conducted among very specific groups such as healthcare workers or university students, which may not be representative of the general population^14,15^.

In this population-based study, we aimed to deliver a comprehensive comparative analysis of self-reported adverse effects up to 12 weeks after receipt of three SARS-CoV-2 vaccines approved in Switzerland in 2021. Further objectives were to examine the general perception and attitudes of individuals regarding SARS-CoV-2 vaccination and their compliance with recommended public health measures. Thereby, we aim to improve our understanding of the adverse health effects experienced following vaccination in the general population to provide an evidence base for future vaccination campaigns in view of the implementation of additional booster vaccinations and updated SARS-CoV-2 vaccines.

## Results

### Cohort characteristics

Of our 575 participants, 323 participants (56.2%) were female, and the median age was 59 years (IQR 41 to 70) (Table 1). 410 (71.3%) participants received an mRNA-based vaccine (36.0% BNT162b2 and 35.3% mRNA-1273) and 165 (28.7%) received a vector-based vaccine (JNJ-78436735). The proportion of participants with a higher level of education (39.9% vs 61.8%) was lower among JNJ-78436735 recipients compared to mRNA vaccine recipients (Supplementary Table 2).

**Table 1:** Demographic and clinical characteristics of the study population.

	BNT162b2 (Pfizer/BioNTech)	mRNA-1273 (Moderna)	JNJ-78436735 (Johnson & Johnson)	Overall
	(*N* = 207)	(*N* = 203)	(*N* = 165)	(*N* = 575)
Age, median (IQR) -in years	59 (36 to 75)	65 (42.5 to 69)	58 (45 to 70)	59 (41 to 70)
Age distribution				
<65 years	106 (51.2%)	99 (48.8%)	103 (62.4%)	308 (53.6%)
≥65 years	101 (48.8%)	104 (51.2%)	62 (37.6%)	267 (46.4%)
Female sex	115 (55.6%)	120 (59.1%)	88 (53.3%)	323 (56.2%)
Presence of at least one self-reported preexisting medical condition	67 (32.4%)	61 (30.0%)	40 (24.2%)	168 (29.2%)
Hypertension	43 (20.8%)	32 (15.8%)	21 (12.7%)	96 (16.7%)
Diabetes	7 (3.4%)	5 (2.5%)	3 (1.8%)	15 (2.6%)
Cardiovascular disease	17 (8.2%)	9 (4.4%)	7 (4.2%)	33 (5.7%)
Respiratory disease	11 (5.3%)	16 (7.9%)	8 (4.9%)	35 (6.1%)
Chronic kidney disease	1 (0.5%)	2 (1.0%)	3 (1.8%)	6 (1.0%)
Current or past malignancy	18 (8.7%)	11 (5.4%)	7 (4.2%)	36 (6.3%)
Immune suppression	3 (1.5%)	1 (0.5%)	3 (1.8%)	7 (1.2%)
Hematologic disease	7 (3.4 %)	8 (3.9%)	5 (3.0%)	20 (3.5%)
Smoking status				
Current smoker	38 (18.4%)	39 (19.5%)	27 (16.9%)	104 (18.3%)
Former smoker	52 (25.1%)	46 (23.0%)	40 (25.0%)	138 (24.4%)
Nonsmoker	117 (56.5%)	115 (57.5%)	93 (58.1%)	325 (57.3%)
Missing	0	3	5	8
Highest educational level				
None or mandatory school	9 (4.4%)	4 (2.0%)	7 (4.3%)	20 (3.5%)
Vocational training or specialized baccalaureate	71 (34.5%)	72 (35.6%)	91 (55.8%)	234 (41.0%)
Higher technical school or college	46 (22.3%)	44 (21.8%)	40 (24.6%)	130 (22.8%)
University	80 (38.8%)	82 (40.6%)	25 (15.3%)	187 (32.7%)
Missing	1	1	2	4
Tested seropositive for anti-SARS-CoV-2 S-IgA prior to vaccination	12 (5.8%)	14 (6.9%)	20 (12.1%)	46 (8.0%)
Tested seropositive for anti-SARS-CoV-2 S-IgG prior to vaccination	15 (7.2%)	19 (9.4%)	24 (14.5%)	58 (10.1%)
Reported positive SARS-CoV-2 test at baseline prior to vaccination	8 (3.9%)	13 (6.4%)	16 (9.7%)	37 (6.4%)
SARS-CoV-2 Infection prior to vaccination (self-reported infection or tested seropositive)	21 (10.1%)	28 (13.8%)	31 (18.8%)	80 (13.9%)
Tested positive for anti-SARS-CoV-2 S-IgA or IgG prior to vaccination with no report of prior SARS-CoV-2 Infection	13 (6.3%)	15 (7.4%)	15 (9.1%)	43 (7.5%)

37 participants (6.4%) reported ever having a positive SARS-CoV-2 test prior to vaccination, with a higher proportion among JNJ-78436735 recipients (9.7% vs 6.4% of mRNA-1273 and 3.9% of BNT162b2 recipients). 19 (9.2%) BNT162b2 recipients, 24 (11.8%) mRNA-1273 recipients and 29 (17.6%) JNJ-78436735 recipients were seropositive for anti-SARS-CoV-2 S-IgA or -IgG before receiving the first vaccination dose.

### Frequency and characteristics of adverse effects

Overall, 79.0% (*N* = 454) of all participants reported at least one adverse effect up to three months following vaccination, with a total of 2233 reported adverse effects. The highest proportion of participants with adverse effects after vaccination was among mRNA-1273 recipients (88.7%, *N* = 180) compared to BNT162b2 (77.3%, *N* = 160) and JNJ-78436735 (69.1%, *N* = 114) recipients. Based on a multivariable logistic regression model, we found strong to very strong evidence that female sex (OR = 2.36 (95% CI: 1.5 to 3.7), *p* < 0.001), higher education levels (vs. none or mandatory school, OR = 4.2 (1.5 to 11.4), *p* = 0.005), receiving mRNA-1273 (vs. BNT162b2, OR = 2.2 (1.2 to 3.8), *p* = 0.008) and SARS-CoV-2 infections prior to vaccination (OR = 2.5 (1.2 to 5.9), *p* = 0.018) were associated with adverse effect reports. We found no evidence that JNJ-78436735 (vs. BNT162b2), preexisting medical conditions, smoking status, and low opinion (opinion value <50) about vaccination and younger age (<65 vs. ≥65 years) were associated with adverse effects (Supplementary Table 3).

**Fig. 1:**
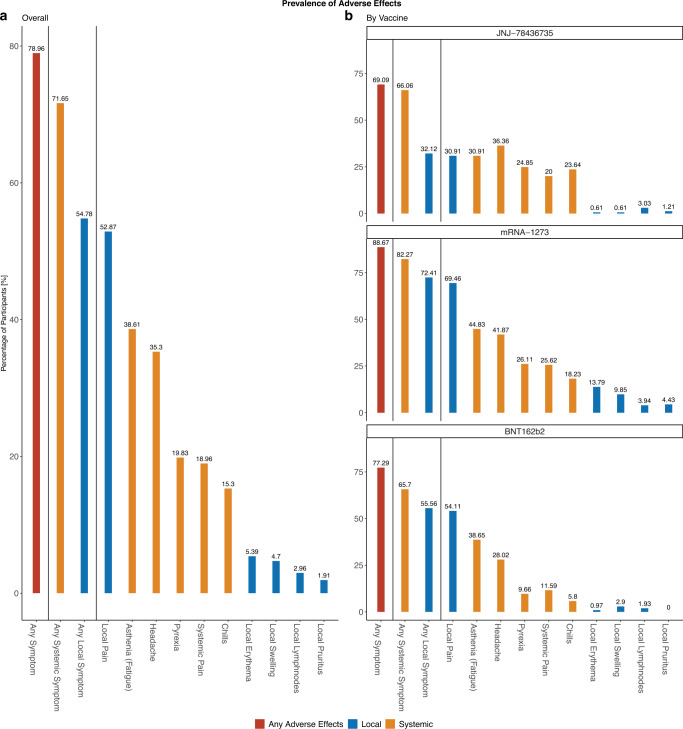
Frequency of any, local and systemic adverse effects. **a** shows the five most common systemic and local adverse effects in the overall sample (*N* = 575). **b** shows the most common adverse effects by vaccine type (BNT162b2 *N* = 208, mRNA-1273 *N* = 203, JNJ-78436735 *N* = 164).

More participants reported systemic (71.7%, *N* = 412) than local adverse effects (54.8%, *N* = 315) (Fig. 1a). Among mRNA vaccine recipients, the proportion of systemic among all adverse effects increased after the 2^nd^ dose (63.9% to 77.2% in BNT162b2 and 59.1% to 78.0% in mRNA-1273 recipients, Supplementary Table 4). The most common adverse effect mentioned by mRNA vaccine recipients was local pain (54.1% of BNT162b2 and 69.5% of mRNA-1273 recipients), followed by asthenia (fatigue; 38.7% of BNT162b2 and 44.8% of mRNA-1273 recipients) (Fig. 1b). JNJ-78436735 recipients most frequently reported headache (36.4%), followed by local pain (30.9%) and asthenia (30.9%) (Fig. 1b). Other commonly reported adverse effects included nausea, vertigo, and sore throat (all >5%). Of all participants, 0.4% (*n* = 2; one BNT162b2 and one mRNA-1273 recipient) reported allergic reactions. Adverse effects affecting menstruation were reported by 5 out of 47 (10.6%) female participants younger than 50 among BNT162b2 recipients, 4 out of 42 (9.5%) among mRNA-1273 recipients, and 2 out of 31 (6.5%) among JNJ-78436735 recipients (six participants reported cycle irregularities, three heavy menstrual bleeding, three intermenstrual bleeding). Tachycardia or palpitations were reported by seven (1.2%) participants, four mRNA-1273, two JNJ-78436735, and one BNT162b2 recipient. One BNT162b2 recipient reported pericardial effusion and atrial fibrillation after the second dose. No thromboembolic events were reported by any of the participants (Supplementary Table 5 for all adverse effects).

**Fig. 2:**
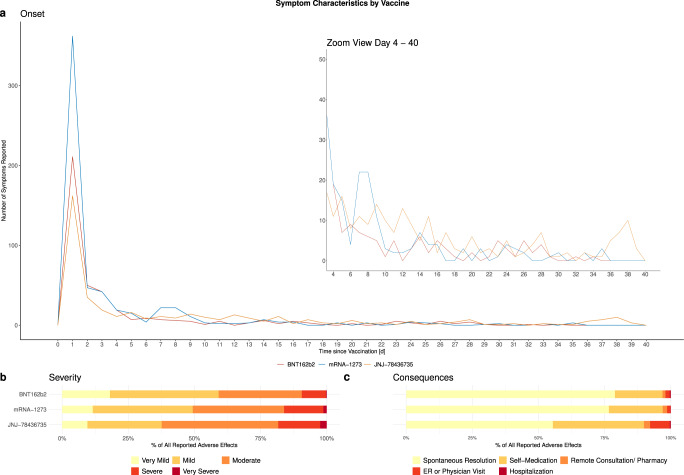
Characteristics of self-reported adverse effects. **a** shows the time of adverse effect onset by the vaccine, **b** shows the perceived adverse effect severity by the vaccine and **c** shows the consequences of adverse effects by the vaccine. ER Emergency Room.

Most adverse effects (83.9%) occurred in the first week following vaccination, 67.9% within 24 h. Participants reported that adverse effects lasted for 3.9 days on average, and most resolved within 3 (76.3%) days. Asthenia, extremity pain, and cough were most frequently reported to last longer than a week. Adverse effect onset and duration were similar across the three vaccines (Fig. 2a, Supplementary Fig. 2).

The perceived severity of most adverse effects was very mild to mild (49.1%) or moderate (36.2%). Meanwhile, 14.7% were described as severe (13.4%) or very severe (1.3%), with the highest proportion of severe to very severe adverse effects reported after JNJ-78436735 (18.3% vs 16.1% after mRNA-1273, 9.4% after BNT162b2) (Fig. 2b, Supplementary Table 6). Asthenia (13.1%), headache (12.8%), and pain (9.4%) were mostly reported as severe or very severe adverse effects. Hospitalization due to reported adverse effects was reported by 0.7% (*n* = 4) of participants (two BNT162b2 recipients with loss of consciousness and bullous pemphigoid, one mRNA-1273 recipient with retinal detachment, and one JNJ-78436735 recipient with meningitis).

Most reported adverse effects resolved spontaneously (Fig. 2c). However, participants reported using self-prescribed medications (e.g., Paracetamol or Ibuprofen) or seeking consultation with a healthcare provider for 448 (20.1%) and 96 (4.3%) of the adverse effects, respectively.

### Perceptions of vaccination and compliance with recommended public health measures

**Fig. 3:**
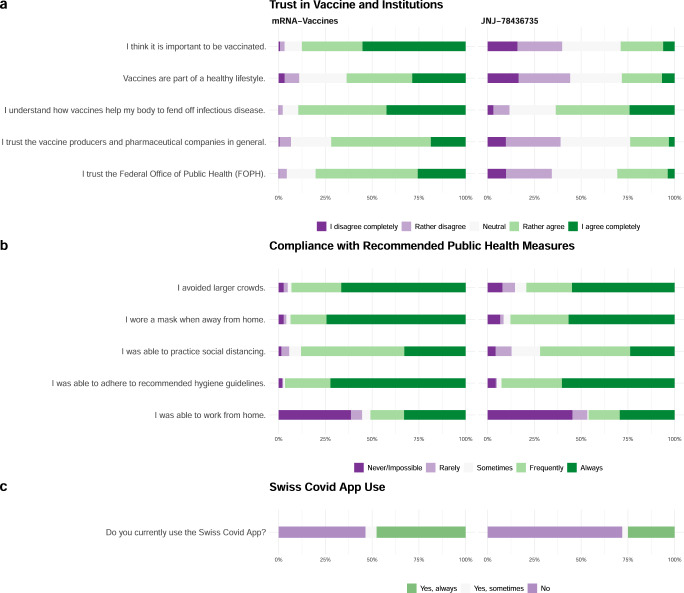
Perception of vaccination and compliance with recommended public health measures. Panel **a** shows the attitude of mRNA and JNJ-78436735 vaccine recipients toward vaccination, pharmaceutical companies and public health authorities. Panel **b** shows self-reported compliance of mRNA and JNJ-78436735 vaccine recipients to recommended public health measures. Panel **c** shows self-reported use of Swiss COVID App among mRNA and JNJ-78436735 vaccine recipients.

A higher proportion of mRNA vaccine recipients (87.5%) agreed completely or in part with the statement that it was important to be vaccinated compared to 28.5% of JNJ-78436735 recipients (Fig. 3a). Similarly, more mRNA vaccine recipients felt that vaccines were part of a healthy lifestyle (63.6% vs. 28.9% of JNJ-78436735 recipients). Trust in public health authorities (80.2% vs. 30.3%) and pharmaceutical companies (71.7% vs. 23.6%) was higher among mRNA vaccine recipients compared to JNJ-78436735 recipients. Both groups felt they had sufficient understanding of how the vaccine helped the body fend off infectious diseases (89.3% mRNA vaccine recipients vs. 62.4% JNJ-78436735 recipients) and reported similar compliance with recommended public health measures (Fig. 3b). Use of the SwissCovid digital proximity tracing app was higher among mRNA vaccine recipients compared to JNJ-78436735 recipients (53.2% vs 27.4%) (Fig. 3c).

## Discussion

In this population-based cohort of 575 individuals who received a SARS-CoV-2 vaccine and were followed-up over 12 weeks, participants commonly reported adverse effects, namely local pain, fatigue, headache, and fever. Most adverse effects were mild to moderate and resolved within three days. Allergic reactions (0.4%) and adverse effects requiring hospitalization (0.7%) were rare. Around 9% of female participants younger than 50 reported menstrual cycle changes, more frequently among mRNA vaccine recipients. Female sex, receiving mRNA-1273, higher education and SARS-CoV-2 infections prior to vaccination were associated with experiencing adverse effects. JNJ-78436735 recipients less frequently perceived vaccination to be important and had lower trust in public health authorities and pharmaceutical companies compared to mRNA vaccine recipients. There were no differences between vaccine groups in compliance with preventive public health measures.

Our results on the prevalence and severity of adverse effects are in line with previously reported data from RCTs and other observational studies^7,8,14,16–18^. In an online survey among individuals vaccinated with either BNT162b2, mRNA-1273, or JNJ-78436735, Beatty et al. reported that 80.3% of participants experienced adverse effects, with comparable estimates for each vaccine type^14^. Our data also matches the prevalence published in the RCTs for each vaccine individually^6–8^. The proportion of adverse effects that were self-reported as severe or required hospitalization in our study (14.7%) was well below that of Swiss and European governmental surveillance systems (37.9% in Swiss ElViS)^9,10^. US surveillance reports also stated higher estimates of serious adverse events based on hospitalization rates, serious illness and deaths (9.2% vs. our 0.7%)^11^. These higher estimates from governmental reporting systems are likely related to the underreporting of mild symptoms and underscore the importance of “real-world” data^12,13^. There is wide variation in reports on prevalence of anaphylaxis and severe allergic reactions ranging from 0.03% to 3%, due to differing definitions^14,19,20^. In our study, two (0.4%) participants reported allergic reactions, without the need to consult a medical professional.

Our follow-up over 12 weeks allowed us to assess adverse effects that occur with some delay, such as menstrual changes which were reported in 9% of female participants younger than 50 years. Few studies have described menstrual irregularities following SARS-CoV-2 vaccination with prevalence ranging between 0.3% and 46%^21,22^. This large variability and the high prevalence (37.8%) of menstrual irregularities in the general population regardless of vaccination underscore the challenge of attributing changes in the menstrual cycle to vaccination^23^. Further research is needed on the influence of SARS-CoV-2 vaccination on menstruation and the general impact of vaccination on female recipients, as we and others observed that female recipients were generally more likely to experience adverse effects^24–27^.

We also found that participants who reported SARS-CoV-2 infections prior to vaccination and received mRNA-1273 were approximately two times more likely to report adverse effects^11,14,18^. These findings have also been reported by others and may be due to increased immunogenicity among these groups^14,15^.

mRNA vaccine recipients trusted vaccines and public health authorities in general and thus were mostly motivated to be vaccinated as soon as SARS-CoV-2 vaccines became available. JNJ-78436735 recipients were more hesitant and waited for the vector-based JNJ-78436735’s introduction to Switzerland, resulting in a higher proportion of individuals with infections prior to vaccination compared to mRNA vaccine recipients. Other studies described these concerns about the rapid development of mRNA vaccines and fears of adverse effects, as well as lack of trust or confidence in governments and their recommendations, to be among the main drivers of vaccine hesitancy and waiting for non-mRNA-based vaccines or other alternatives^28–33^. General skepticism and the presence of nocebo effects, as demonstrated by Amanzio et al., may have translated into a higher proportion of JNJ-78436735 recipients perceiving adverse effects as severe^30,34,35^. Increasing awareness of these nocebo responses and using positive framing around the low risk of severe adverse effects may contribute to improving vaccine acceptance.

This study provided evidence from a representative cohort recruited from the general population and followed-up over a 12-week period. Data collected through symptom diaries and adverse effect coding according to MedDRA terms generated a comprehensive dataset allowing a comparative analysis of three common SARS-CoV-2 vaccines. However, there are several limitations to our study. First, self-selection bias may have occurred if individuals who are more health literate, less hesitant, or who have less negative perceptions regarding vaccination, were more likely to participate in our study. Overall, this may have led to an overestimation of trust in public health authorities and more positive perceptions of vaccines. However, we consider the data on the prevalence of adverse effects as broadly representative. Furthermore, since we only included individuals who received a SARS-CoV-2 vaccine, our findings regarding vaccine trust and perceptions may not be fully generalizable to individuals who decided against vaccination. However, since our study included those who delayed vaccination until the JNJ-78436735 became available in Switzerland, we believe our study still provides insights into those who are vaccine hesitant to a certain extent. Second, since mRNA and JNJ-78436735 vaccines were rolled-out sequentially in Switzerland, the recruitment timeframes of the two vaccine groups were different (mRNA recipients: March 2021 to July 2021; JNJ-78436735 recipients: October 2021 to January 2022). We cannot exclude that some of the differences in results are related to temporal changes in participants’ perceptions that we could not account for in our analyses. Accumulating evidence on SARS-CoV-2 vaccines may have led to better perceptions, leading to an underestimation of the differences between the vaccine groups. On the other hand, discourse around vaccination and the implementation of vaccine certificates may have led to worse perceptions and consequently an overestimation of the differences. Similarly, there may have been an inclination to underreport (due to increasing evidence on vaccine safety) or overreport certain adverse effects (due to increased public discourse) over time. Overall, it is thus difficult to estimate the direction of bias arising from these potential changes over time. However, this needs to be considered when interpreting the findings of the study. Third, our data is self-reported. While this allows for an accurate description of vaccine recipients’ experiences, it is subjective and no verification of the relation of adverse effects and vaccination by a healthcare provider was possible. Fourth, the absolute numbers of reports for some adverse effects when analyzed individually are relatively small (e.g., menstrual changes). Fifth, participants were followed up for 12 weeks after the first vaccine dose. Individuals who were vaccinated with an mRNA vaccine also had a second dose 3–4 weeks after the first dose, while those who received the JNJ-78436735 vaccine received only one dose in total. Consequently, the total follow-up after completion of the vaccination schedule differed between the two groups. The shorter follow-up time in the mRNA vaccine group may have led to a possible underestimation of the frequency of adverse effects compared to those who received a JNJ-78436735 vaccine. However, since only very few adverse effects were observed after two weeks, we consider it unlikely that this difference in the follow-up time had a significant influence on the estimates. Sixth, we cannot exclude that some adverse effects were related to an undiagnosed SARS-CoV-2 infection rather than vaccination. Although we excluded any adverse effects reported three days before and at any timepoint after a self-reported positive SARS-CoV-2 test, some infections may have gone undetected if the participants attributed any related symptoms to the vaccine or did not get tested. Since the effectiveness of JNJ-78436735 in preventing infections is lower than mRNA vaccines and the recruitment of JNJ-78436735 recipients began less than three months before the onset of the Omicron wave in Switzerland, we would expect a higher proportion of the reported adverse effects to potentially be secondary to infection among JNJ-78436735 recipients compared to mRNA vaccine recipients if above is the case^36^. This would imply a possible overestimation of the prevalence of adverse effects among those receiving JNJ-78436735 and, given the higher prevalence of adverse effects among mRNA vaccine recipients, an underestimation of the differences in adverse effects prevalence between JNJ-78436735 and the mRNA vaccines. Finally, our analysis was restricted to the primary vaccination series of the three SARS-CoV-2 vaccines approved in Switzerland at the time of study conduct. Further research on adverse effects occurring after booster vaccinations, other types of vaccines, and combinations of different vaccines are needed.

In conclusion, this study demonstrates the safety of three SARS-CoV-2 vaccines in a representative population-based cohort and provides real-world estimates of the prevalence of adverse effects after vaccination. Thereby, we importantly extend the evidence base for healthcare providers to answer many of the questions of individuals seeking vaccination. While further evidence on adverse effects after booster vaccination and other vaccine types is required, our study suggests that transparent communication regarding adverse effects and building trust in public health authorities are pivotal to future vaccination campaigns’ success.

## Methods

### Swiss vaccination context

In Switzerland, three SARS-CoV-2 vaccines were approved and made available in 2021. BNT162b2 vaccine was available as of late December 2020 and mRNA-1273 as of late January 2021, whereas JNJ-78436735 (Johnson & Johnson) only became available as of October 2021. Until June 2021, vaccination was made incrementally available to the population according to defined target vaccination groups^37,38^, with priority given to older populations, at-risk populations and healthcare personnel. In the Canton of Zurich, individuals wanting to receive a vaccination were assigned to one of the target vaccination groups by the Department of Health and referred to the University of Zurich (UZH) reference vaccination center for receipt of the vaccine. In the primary vaccination series, individuals vaccinated with either of the mRNA vaccines received two doses 3–4 weeks apart, while those vaccinated with JNJ-78436735 received one dose, as per recommendations^39–41^. To note that individuals who presented for the BNT162b2 and mRNA-1273 vaccines at the vaccination center could not choose between the two types of mRNA vaccines; they were given the type of mRNA vaccine which was available at the center on that specific day. Individuals receiving JNJ-78436735 specifically asked for receiving this vaccine rather than mRNA vaccines.

### Study design, participants, and recruitment

This study is based on the Zurich SARS-CoV-2 Vaccine Cohort, an ongoing prospective population-based observational study. We recruited participants from individuals registered for primary SARS-CoV-2 vaccination at the UZH vaccination center in Switzerland. Individuals receiving BNT162b2 or mRNA-1273 vaccines were recruited between March 10, 2021, and July 21, 2021 while participants receiving JNJ-78436735 were recruited between October 20, 2021 and January 27, 2022.

**Fig. 4:**
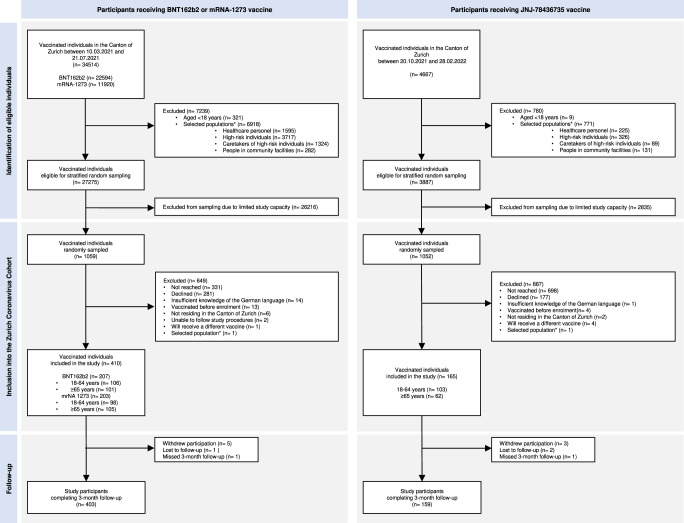
Recruitment of study cohort (*as defined by the Canton of Zurich’s vaccination guidelines^42,43^). The left panel shows the recruitment flowchart of participants receiving BNT162b2 or mRNA-1273 vaccine and the right panel shows the recruitment flowchart of participants receiving JNJ-78436735 vaccine.

We screened all individuals scheduled to receive one of the three SARS-CoV-2 vaccines at the center for study eligibility on a daily basis. Eligibility criteria were being 18 years or older, being able to follow study procedures, having sufficient knowledge of the German language and residing in the Canton of Zurich. We excluded individuals who had already received a first dose of a SARS-CoV-2 vaccine. We then daily selected an age-stratified (18–64 years, 65 years or older) random sample was selected separately for each approved vaccine from all eligible individuals belonging to the following target vaccination groups as defined by the Canton of Zurich:^42,43^ “Over 75 years”, “over 65 years”, “between 50–64 years”, and “between 18–49 years”. We excluded individuals belonging to groups specific for “healthcare workers”, “caretakers of high-risk patients”, “individuals living in communal facilities”, and “individuals with the highest risk diseases” (i.e., those at an advanced stage of certain diseases such as decompensated heart or liver failure) to ensure that our sample was as representative of the general population as possible (Fig. 4)^37,38,42,43^. Randomly selected individuals were then invited to participate in our study. We obtained written informed consent from all participants. We were unable to reach the desired sample size for JNJ-78436735 recipients 65 years or older, due to limited demand.

The study protocol was prospectively registered on the International Standard Randomized Controlled Trial Number Registry (ISRCTN 15499304) and approved by the ethics committee of the Canton of Zurich (BASEC 2021-00273).

### Data sources and measurements

Upon enrollment, all participants completed a baseline questionnaire including questions on their sociodemographics, pre-existing self-reported medical conditions (i.e., hypertension, diabetes mellitus, cardiovascular disease, respiratory disease, chronic renal disease, current or past malignancy, or immune suppression), smoking history, SARS-CoV-2 related information such as prior infections, perceptions and attitudes regarding vaccination, trust in public health authorities and pharmaceutical companies, and compliance with recommended public health measures. Perception, attitude, and compliance questions were collected using a numerical scale (0 “Very low opinion”, to 100 “Very high opinion” for perception), or a 5-item Likert scale (ranging from “Strongly disagree” to “Strongly agree” for trust-related statements, and from “Never/Impossible” to “Always” for statements on compliance with public health measures). Compliance with public health measures also included use of the “SwissCovid” digital proximity tracing app, a publicly available mobile app launched by public health authorities in June 2020 to complement manual contact tracing^44^. We pooled BNT162b2 and mRNA-1273 recipients into one “mRNA vaccine” group since they expressed similar perceptions towards vaccination and compliance with recommended measures (Supplementary Fig. 1).

At the time of enrollment into the study, we provided participants with a paper symptom diary and instructed them to prospectively record any adverse effects that they experience at any point up to 12 weeks after vaccination, as free text. The information recorded in the symptom diary included the start and end dates of the adverse effects, perceived severity (on a 5-item Likert scale, ranging from “Very mild” to “Very severe”), and their consequences (i.e., self-medication, need for healthcare services, or hospitalizations). We reminded participants to fill the symptom diaries during their follow-up visits (4 and 6 weeks after vaccination) and collected the symptom diaries at the 12-week follow-up visit. Participants received additional electronic questionnaires at 4, 6, and 12 weeks after vaccination, in which they were asked to report any positive SARS-CoV-2 polymerase chain reaction (PCR) or rapid antigen tests. We excluded all reported adverse effects starting within three days before and at any timepoint after positive SARS-CoV-2 tests to ensure that reported symptoms were related to vaccination rather than infection. To determine the proportion of participants with past SARS-CoV-2 infection, we measured participants’ anti-SARS-CoV-2 Spike (S)-IgA and IgG antibodies at baseline using a highly sensitive and specific Luminex technology-based assay^45^.

We collected and managed all study data using the Research Electronic Data Capture (REDCap) system^46,47^.

### Outcomes

Our primary outcomes included period prevalence, onset, duration, and severity of self-reported adverse effects over 12 weeks after the first vaccine dose, with a specific focus on the proportion of participants reporting allergic reactions, menstrual irregularities, or cardiac adverse effects, or requiring hospitalization. Secondary outcomes included risk factors associated with adverse effect reports, general perceptions and attitudes regarding vaccination, trust in public health authorities and pharmaceutical companies, and compliance with recommended public health measures.

### Statistical analysis

We descriptively analyzed the characteristics and outcomes of interest for the overall cohort and for each of the three vaccine groups. Continuous variables are reported as median with interquartile range (IQR); categorical or ordinal variables as frequencies (N) and percentages (%). We coded adverse effect data reported by participants in the symptom diary according to the Medical Dictionary for Regulatory Activities (MedDRA) hierarchical terminology (Supplementary Table 1)^48^. The self-reported adverse effects were translated from German to the closest matching MedDRA “low level term”. All corresponding higher-level terms were included in the database, and the highest level of coding was added, labeling each adverse effect either as “local” or “systemic”. We explored associations of several predictor variables on the outcome of reporting one or more adverse effects using a multivariable logistic regression model. Separate models were run for each predictor variable and all models were adjusted for age, sex, body mass index, vaccine type, and education. These variables were included based on findings from other studies^14,49–52^. Results are presented as odds ratios (OR) with their 95% confidence intervals (CI) and two-sided *p*-value. All analyses were performed using R (version 4.1.2).

### Reporting summary

Further information on research design is available in the Nature Research Reporting Summary linked to this article.

## Supplementary information

Supplementary Material

Dataset 1

REPORTING SUMMARY

## Data Availability

Deidentified individual participant data underlying the findings of this study are available as supplementary material.

## Code availability

The analysis code (R programming software) that can be used to replicate the findings of this study is available as supplementary material.
