# Supplementary material for: Adverse effects, perceptions and attitudes related to BNT162b2, mRNA-1273 or JNJ-78436735 SARS-CoV-2 vaccines: Population-based cohort
Source: NPJ Vaccines. 2023 Apr 24;8:61. doi: 10.1038/s41541-023-00657-3 (PMC10123463; doi:10.1038/s41541-023-00657-3)
Supplement: Supplementary file 1 — Supplementary Material [file 41541_2023_657_MOESM1_ESM.pdf]

Supplementary Material

Supplementary Figure 1. Answers to trust related questions among mRNA vaccine recipients

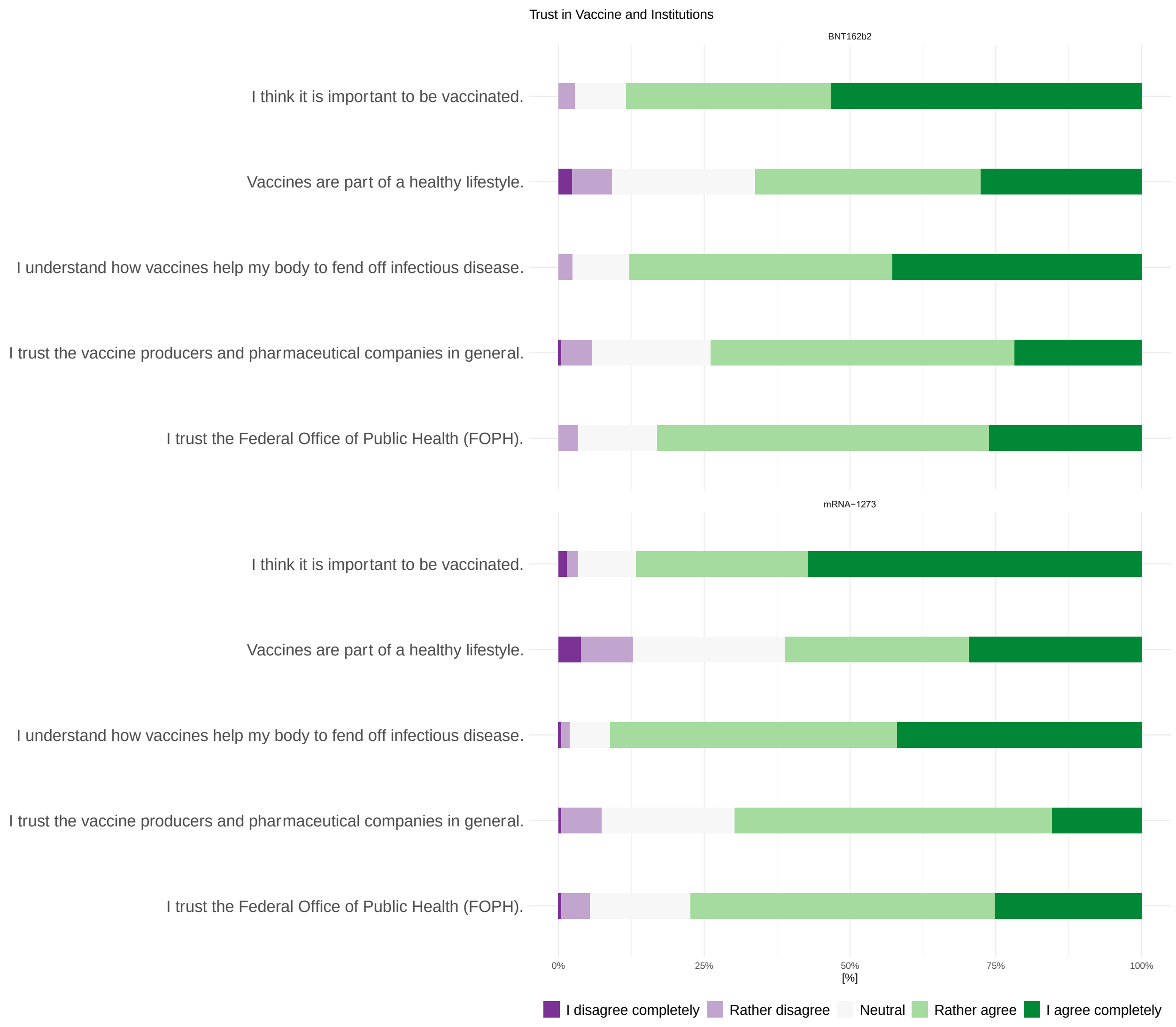

Supplementary Table 1. MedDRA coding hierarchy (NEC= not elsewhere classified)

System Organ Class	High Level Group Term	High Level Term	Preferred Term	Low Level Term
blood and lymphatic system disorders	spleen, lymphatic and reticuloendothelial system disorders	lymphatic system disorders NEC	lymphadenopathy	axillary lymph nodes enlarged
				lymph nodes cervical swollen
				lymph nodes enlarged
cardiac disorders	cardiac arrhythmias	supraventricular arrhythmias	atrial fibrillation	atrial fibrillation
		rate and rhythm disorders NEC	tachycardia	tachycardia
	cardiac disorders, signs, and symptoms NEC	cardiac signs and symptoms NEC	palpitations	palpitations
	pericardial disorders	pericardial disorders NEC	pericardial effusion	pericardial effusion
ear and labyrinth disorders	aural disorders NEC	ear disorders NEC	ear inflammation	ear inflammation
			ear pain	ear pain
eye disorders	eye disorders NEC	lacrimation disorders	dry eye	dry eyes
			lacrimation increased	watering eyes
		ocular disorders NEC	ocular discomfort	sensation of pressure in eye

System Organ Class	High Level Group Term	High Level Term	Preferred Term	Low Level Term
	ocular infections, irritations and inflammations	conjunctival infections, irritations and inflammations	conjunctivitis	conjunctivitis
		lid, lash and lacrimal infections, irritations and inflammations	blepharitis	blepharitis
		ocular infections, inflammations and associated manifestations	eye irritations	burning eyes
			ocular hyperemia	eye red
	ocular structural change, deposit and degeneration NEC	retinal structural change, deposit and degeneration	retinal detachment	retinal detachment
	vision disorders	visual disorders NEC	vision blurred	blurred vision
		visual impairment and blindness (excluding color blindness)	visual impairment	visual impairment
gastrointestinal disorders	dental and gingival conditions	gingival Infections	gingivitis	gingivitis
		gingival disorders, signs and symptoms NEC	Non-infective gingivitis	sores gum
		dental pain and sensation disorders	toothache	tooth pain

System Organ Class	High Level Group Term	High Level Term	Preferred Term	Low Level Term
	gastrointestinal infections	gastric and gastrointestinal infections	gastroenteritis viral	stomach flu
	gastrointestinal motility and defecation conditions	diarrhea (excluding infective)	diarrhea	diarrhea
	gastrointestinal signs and symptoms	dyspeptic signs and symptoms	dyspepsia	digestion impaired
				stomach burning sensation
		flatulence, bloating and distension	abdominal distension	abdominal bloating
			flatulence	flatulence
		gastrointestinal and abdominal pains (excluding oral and throat)	abdominal pain	abdominal cramps
				abdominal pain
			abdominal pain upper	stomachache
				stomach cramps
				stomach pain
		gastrointestinal signs and symptoms NEC	abdominal discomfort	abdominal discomfort
				stomach discomfort
			breath odor	bad breath

System Organ Class	High Level Group Term	High Level Term	Preferred Term	Low Level Term
			odynophagia	swallowing painful
		nausea and vomiting symptoms	nausea	nausea
			vomiting	vomiting
	gastrointestinal vascular conditions	hemorrhoids and gastrointestinal varices (excluding oesophageal)	haemorrhoidal haemorrhage	haemorrhoidal bleeding
	oral soft tissue conditions	oral soft tissue signs and symptoms	hypoaesthesia oral	numbness of tongue
			lip discoloration	lip discolouration
			oral pain	sensitive mouth
	saliva gland conditions	oral dryness and saliva altered	dry mouth	dry mouth
	tongue conditions	tongue signs and symptoms	tongue discolouration	tongue white
general disorders and administration site conditions	administration site reactions	injection site reactions	injection site erythema	injection site redness
			injection site pruritus	injection site itching
		vaccination site reactions	vaccination site discolouration	vaccination site discolouration
			vaccination site haematoma	vaccination site hematoma

System Organ Class	High Level Group Term	High Level Term	Preferred Term	Low Level Term
			vaccination site inflammation	vaccination site inflammation
			vaccination site irritation	vaccination site irritation
			vaccination site movement impairment	vaccination site movement impairment
			vaccination site pain	vaccination site pain
			vaccination site rash	vaccination site rash
			vaccination site swelling	vaccination site swelling
			vaccination site tenderness	vaccination site tenderness
			vaccination site warmth	vaccination site warmth
	body temperature conditions	febrile disorders	pyrexia	fever
				feverish
	general system disorders NEC	asthenic conditions	asthenia	energy decreased
				fatigue
				feeling of weakness

System Organ Class	High Level Group Term	High Level Term	Preferred Term	Low Level Term
				feelings of weakness
				weakness
			listlessness	listlessness
			malaise	feeling unwell
				unwell
		feelings and sensations NEC	chills	chills
			decreased appetite	appetite absent
			feeling abnormal	feeling dazed
			feeling cold	feeling cold
			feeling hot	feeling hot
				feeling of warmth
				sensation of heat
			hot flush	feeling of hot flushes
			hunger	feeling hungry
			irritability	feeling irritated
			peripheral coldness	cold extremities
				cold feet
			temperature intolerance	heat sensitivity
			thirst	thirst
			balance disorder	unsteadiness

System Organ Class	High Level Group Term	High Level Term	Preferred Term	Low Level Term
		general signs and symptoms NEC	hot flushes	hot flashes
				hot flushes
			hyperhidrosis	excess sweating
				heavy sweating
				sweating
				sweating attack
			influenza like illness	flu-like symptoms
			night sweats	night sweats
			peripheral swelling	swelling arm
				swelling of legs
			swelling	swelling
		oedema NEC	oedema peripheral	leg edema
		pain and discomfort NEC	axillary pain	armpit pain
			chest discomfort	chest pressure
			chest pain	chest burning
				chest pain
				thorax pain
			pain	general body pain
				pain
immune system disorders	allergic conditions	allergic conditions NEC	hypersensitivity	allergic reaction
		angioedemas	swollen tongue	swollen tongue

System Organ Class	High Level Group Term	High Level Term	Preferred Term	Low Level Term
		urticarias	urticaria	urticaria
infections and infestations	viral infectious disorders	herpes viral infections	oral herpes	cold sores
				cold sores lip
				herpes labialis
investigations	cardiac and vascular investigations (excl enzyme tests)	heart rate and pulse investigations	heart rate increased	pulse rate increased
	physical examination and organ system status topics	physical examination procedures and organ system status	body temperature increased	temperature elevation
metabolism and nutrition disorders	electrolyte and fluid balance conditions	total fluid volume increase	oedema	oedematous weight gain
musculoskeletal and connective tissue disorders	bone disorders (excl congenital and fractures)	bone related signs and symptoms	bone pain	bone pain
		bone disorders NEC	exostosis	bone spur
	joint disorders	joint related signs and symptoms	arthralgia	joint pain
				knee pain
				pain in joint
				pain in joint involving hand
				painful joints
				shoulder pain

System Organ Class	High Level Group Term	High Level Term	Preferred Term	Low Level Term
		osteoarthropathies	osteoarthritis	arthrosis
				gonarthrosis
	muscle disorders	muscle pains	myalgia	muscle pain
				muscle soreness
				tenderness muscle
		muscle related signs and symptoms NEC	muscle spasms	leg cramps
				muscle cramps
			muscle swelling	muscle swelling
			muscle twitching	muscle twitching
		muscle weakness conditions	muscular weakness	muscle weakness lower limb
		myopathies	myopathy	myopathy
	musculoskeletal and connective tissue disorders NEC	musculoskeletal and connective tissue conditions NEC	musculoskeletal stiffness	neck stiffness
				stiffness shoulder
		musculoskeletal and connective tissue pain and discomfort	back pain	back pain
				lumbago
				muscular back pain
			flank pain	flank pain
			limb discomfort	feeling heavy in arms and legs

System Organ Class	High Level Group Term	High Level Term	Preferred Term	Low Level Term
				heavy feeling in arms and legs
			musculoskeletal pain	pain neck/ shoulder
			neck pain	neck pain
			pain in extremity	leg pain
				pain foot
				pain in fingers
				pain in thumb
				pain in toe
				painful arm
				painful feet
				painful hand
	tendon, ligament and cartilage disorders	tendon disorders	tenosynovitis	tendovaginitis
nervous system disorders	central nervous system infections and inflammations	meningitis NEC	meningitis	meningitis
	cranial nerve disorders (excl neoplasms)	olfactory nerve disorders	anosmia	smell loss
		auditory nerve disorders	tinnitus	subjective tinnitus
	headaches	headaches NEC	headache	headache

System Organ Class	High Level Group Term	High Level Term	Preferred Term	Low Level Term
				throbbing headache
		migraine headaches	migraine	migraine
				migraine with aura
	movement disorders (incl parkinsonism)	tremor (excl congenital)	tremor	shaking of hands
	neurological disorders NEC	disturbances in consciousness NEC	loss of consciousness	Consciousness loss of
		neurological signs and symptoms NEC	head discomfort	head pressure
		paraesthesia and dysaesthesia	paraesthesia	tingling sensation
		sensory abnormalities NEC	sensory loss	loss of sensation
		vertigos NEC	vertigo	vertigo
psychiatric disorders	cognitive and attention disorders and disturbances	cognitive and attention disorders and disturbances NEC	disturbance in attention	poor concentration
	deliria (incl confusion)	confusion and disorientation	confusional state	confusion
	dementia and amnestic conditions	amnestic symptoms	memory impairment	forgetfulness

System Organ Class	High Level Group Term	High Level Term	Preferred Term	Low Level Term
	depressed mood disorders and disturbances	depressive disorders	depression	depression
		mood alterations with depressive symptoms	depressed mood	depressed mood
	mood disorders and disturbances NEC	emotional and mood disturbances NEC	euphoric mood	euphoria
	sleep disorders and disturbances	disturbances in initiating and maintaining sleep	insomnia	sleeplessness
		dyssomnias	poor quality sleep	poor sleep
				sleep restless
		sleep disorder NEC	sleep disorder	disorder sleep
				sleep problem
	somatic symptom and related disorders	somatic symptom disorders	conversion disorder	Functional neurological symptom disorder
renal and urinary disorders	bladder and bladder neck disorders (excl calculi)	bladder disorders NEC	bladder disorder	bladder disorder
		bladder infections and inflammations	cystitis haemorrhagic	cystitis hemorrhagic
	urinary tract signs and symptoms	bladder and urethral symptoms	dysuria	painful urination
			incontinence	incontinence
			micturition urgency	urgency urination

System Organ Class	High Level Group Term	High Level Term	Preferred Term	Low Level Term
reproductive system and breast disorders	breast disorders	breast signs and symptoms	breast pain	mastodynia
	menstrual cycle and uterine bleeding disorders	menstrual and uterine bleeding NEC	intermenstrual bleeding	spotting between menses
		menstruation with increased bleeding	heavy menstrual bleeding	heavy menstrual bleeding
			menstruation irregular	menstrual irregularity
				menstruation irregular
	vulvovaginal disorders (excl infections and inflammations)	vulvovaginal disorders NEC	vaginal haemorrhage	spotting vaginal
respiratory, thoracic and mediastinal disorders	pleural disorders	pneumothorax and pleural effusions NEC	pleural effusion	pleural effusion
	respiratory disorders NEC	breathing abnormalities	dyspnoea	difficulty breathing
		coughing and associated symptoms	cough	cough
	respiratory tract infections	upper respiratory tract infections NEC	laryngitis	laryngitis
			nasopharyngitis	cold symptoms
			sinusitis	sinusitis

System Organ Class	High Level Group Term	High Level Term	Preferred Term	Low Level Term
		viral upper respiratory tract infections	influenza	flu symptoms
	respiratory tract signs and symptoms	lower respiratory tract signs and symptoms	hiccups	hiccups
		upper respiratory signs and symptoms	aphonia	loss of voice
			dry throat	dry throat
			speech disorder	disorder speech
		upper respiratory tract signs and symptoms	oropharyngeal pain	sore throat
			rhinorrhoea	rhinorrhea
			sneezing	sneezing
	upper respiratory tract disorders (excl inflammation)	nasal congestions and inflammation	nasal congestion	nose congestion
			rhinitis allergic	allergic rhinitis
		nasal disorders NEC	epistaxis	nosebleed
skin and subcutaneous tissue disorders	epidermal and dermal conditions	bullous conditions	pemphigoid	bullous pemphigoid
		dermal and epidermal conditions NEC	dry skin	dry skin
			hypoaesthesia	body numbness
			skin burning sensation	skin burning sensation
		erythemas	erythema	redness
				redness of face
				redness of legs

System Organ Class	High Level Group Term	High Level Term	Preferred Term	Low Level Term
		papulosquamous conditions	oral lichen planus	oral lichen planus
		pruritus NEC	pruritus	generalized itching
				itching
		pustular conditions	rash pustular	pustular rash
		rashes, eruptions and exanthems NEC	rash	facial rash
				skin rash
			rash erythematous	red rash
			rash macular	red spotty rash
			rash pruritic	itchy rash
	skin and subcutaneous tissue infections and infestations	skin and subcutaneous tissue bacterial infections	furuncle	boil
		skin and subcutaneous tissue viral infections	herpes zoster	herpes zoster
	skin appendage conditions	alopecias	alopecia	hair loss
		pilar disorders NEC	piloerection	goose bumps
vascular disorders	decreased and nonspecific blood pressure disorders and shock	circulatory collapse and shock	dizziness	dizziness
				light headedness
		vascular hypotensive disorders	hypotension	low blood pressure

System Organ Class	High Level Group Term	High Level Term	Preferred Term	Low Level Term
	vascular hypertensive disorders	vascular hypertensive disorders NEC		hypertension

Supplementary Table 2. Demographic and clinical characteristics of participants, with BNT162b2 and mRNA-1273 combined into mRNA vaccine group.

	**mRNA vaccines (BNT162b2 and mRNA-1273)**	**JNJ-78436735 (Johnson & Johnson)**	**Overall**
	**(N=410)**	**(N=165)**	**(N=575)**
**Age, median (IQR) -in years**	64.5 (39.0 to 71.0)	58.0 (45.0 to 70.0)	59.0 (41.0 to 70.0)
**Age distribution**			
<65 years	205 (50.0%)	103 (62.4%)	308 (53.6%)
≥65 years	205 (50.0%)	62 (37.6%)	267 (46.4%)
**Female sex**	235 (57.3%)	88 (53.3%)	323 (56.2%)
**Presence of at least one preexisting medical condition**	128 (31.2%)	40 (24.2%)	168 (29.2%)
Hypertension	75 (18.3%)	21 (12.7%)	96 (16.7%)
Diabetes	12 (3.0%)	3 (1.8%)	15 (2.6%)
Cardiovascular disease	26 (6.3%)	7 (4.2%)	33 (5.7%)
Respiratory disease	27 (6.5%)	8 (4.8%)	35 (6.1%)
Chronic kidney disease	3 (0.7%)	3 (1.8%)	6 (1.0%)
Current or past malignancy	29 (7.1%)	7 (4.2%)	36 (6.3%)
Immune suppression	4 (1.0%)	3 (1.8%)	7 (1.2%)
Hematologic disease	15 (3.7%)	5 (3.0%)	20 (3.5%)
**Smoking status**			
Current smoker	77 (18.9%)	27 (16.9%)	104 (18.3%)
Former smoker	98 (24.1%)	40 (25.0%)	138 (24.4%)

Nonsmoker	232 (57.0%)	93 (58.1%)	325 (57.3%)
Missing	3	5	8
**Highest educational level**			
None or mandatory school	13 (3.2%)	7 (4.3%)	20 (3.5%)
Vocational training or specialized baccalaureate	143 (35.0%)	91 (55.8%)	234 (41.0%)
Higher technical school or college	90 (22.1%)	40 (24.6%)	130 (22.8%)
University	162 (39.7%)	25 (15.3%)	187 (32.7%)
Missing	2	2	4
**Tested seropositive for anti-SARS-CoV-2 S-IgA prior to vaccination**	26 (6.3%)	20 (12.1%)	46 (8.0%)
**Tested seropositive for anti-SARS-CoV-2 S-IgG prior to vaccination**	34 (8.3%)	24 (14.5%)	58 (10.1%)
**Reported positive SARS-CoV-2 test at baseline prior to vaccination**	21 (5.1%)	16 (9.7%)	37 (6.4%)
**SARS-CoV-2 Infection prior to vaccination (self-reported infection or tested seropositive)**	49 (12.0%)	31 (18.8%)	80 (13.9%)
**Tested positive for anti-SARS-CoV-2 S-IgA or IgG prior to vaccination with no report of prior SARS-CoV-2 Infection**	28 (6.8%)	15 (9.1%)	43 (7.5%)
**Reported adverse effects - no. of participants**	340 (82.9%)	114 (69.1%)	454 (79.0%)

Supplementary Table 3. Factors associated with adverse effect development after SARS-CoV2 vaccination from a multivariable regression analysis. A separate model was ran for each predictor variable, and all were adjusted for age group..

**Characteristic**	**OR (95%CI)**	**p-value**
**Age <65 years (vs. ≥65 years)**	1.40 (0.89 to 2.21)	0.145
**Female sex (vs male)**	2.36 (1.52 to 3.73)	<0.001
**Body mass index (kg/m2)**	0.96 (0.91 to 1.01)	0.146
**Vaccine**		
BNT162b2	1 [Reference]	
mRNA-1273	2.15 (1.24 to 3.81)	0.008
JNJ-78436735	0.68 (0.41 to 1.14)	0.146
**Education**		
None or mandatory school	1 [Reference]	
Vocational training or specialized baccalaureate	4.25 (1.58 to 11.48)	0.004
Higher education	4.18 (1.54 to 11.35)	0.005
**Any preexisting condition (vs. none)**	0.99 (0.57 to 1.73)	0.958
**Smoking Status**		
Non-Smoker	1 [Reference]	
Smoker	0.68 (0.39 to 1.22)	0.189
Former Smoker	1.11 (0.65 to 1.93)	0.705
**Low opinion of vaccination (vs. high)**	0.81 (0.42 to 1.61)	0.536
**SARS-CoV-2 Infection prior to vaccination (self-reported infection or tested seropositive) (vs. none)**	2.54 (1.23 to 5.85)	0.018

Supplementary Table 4. Frequency of systemic and local adverse effects reported by participants after the first and second BNT162b2 and mRNA-1273 dose.

Adverse Effects by Vaccine Type	First dose	Second dose	Overall
**mRNA-1273**	**(N=452)**	**(N=587)**	**(N=1039)**
Local adverse effects	185 (40.9%)	129 (22.0%)	314 (30.2%)
Systemic adverse effects	267 (59.1%)	458 (78.0%)	725 (69.8%)
**BNT162b2**	**(N=319)**	**(N=378)**	**(N=697)**
Local adverse effects	115 (36.1%)	86 (22.8%)	201 (28.8%)
Systemic adverse effects	204 (63.9%)	292 (77.2%)	496 (71.2%)

Supplementary Table 5. Frequency of adverse effects according to MedDRA high level terms in alphabetic order (NEC = not elsewhere classified)

**MedDRA High Level Terms**	**BNT162b2 (N=697)**	**mRNA-1273 (N=1039)**	**JNJ-78436735 (N=661)**	**Overall (N=2397)**
amnestic symptoms	1 (0.1%)	0 (0.0%)	0 (0.0%)	1 (0.0%)
asthenic conditions	115 (16.5%)	132 (12.7%)	72 (10.9%)	319 (13.3%)
bone disorders NEC	1 (0.1%)	0 (0.0%)	0 (0.0%)	1 (0.0%)
bone related signs and symptoms	1 (0.1%)	0 (0.0%)	0 (0.0%)	1 (0.0%)
breathing abnormalities	5 (0.7%)	2 (0.2%)	5 (0.8%)	12 (0.5%)
bullous conditions	2 (0.3%)	0 (0.0%)	0 (0.0%)	2 (0.1%)
cardiac signs and symptoms NEC	2 (0.3%)	3 (0.3%)	1 (0.2%)	6 (0.3%)
circulatory collapse and shock	2 (0.3%)	7 (0.7%)	9 (1.4%)	18 (0.8%)
coughing and associated symptoms	7 (1.0%)	7 (0.7%)	9 (1.4%)	23 (1.0%)
dermal and epidermal conditions NEC	2 (0.3%)	0 (0.0%)	0 (0.0%)	2 (0.1%)
diarrhoea (excl infective)	15 (2.2%)	10 (1.0%)	9 (1.4%)	34 (1.4%)
disturbances in consciousness NEC	1 (0.1%)	0 (0.0%)	0 (0.0%)	1 (0.0%)
disturbances in initiating and maintaining sleep	1 (0.1%)	1 (0.1%)	0 (0.0%)	2 (0.1%)
dyspeptic signs and symptoms	2 (0.3%)	3 (0.3%)	3 (0.5%)	8 (0.3%)
dyssomnias	3 (0.4%)	2 (0.2%)	5 (0.8%)	10 (0.4%)
ear disorders NEC	2 (0.3%)	5 (0.5%)	3 (0.5%)	10 (0.4%)
erythemas	1 (0.1%)	3 (0.3%)	0 (0.0%)	4 (0.2%)
febrile disorders	20 (2.9%)	63 (6.1%)	55 (8.3%)	138 (5.8%)
feelings and sensations NEC	23 (3.3%)	54 (5.2%)	53 (8.0%)	130 (5.4%)
gastric and gastroenteric infections	1 (0.1%)	0 (0.0%)	0 (0.0%)	1 (0.0%)

gastrointestinal and abdominal pains (excl oral and throat)	5 (0.7%)	8 (0.8%)	1 (0.2%)	14 (0.6%)
gastrointestinal signs and symptoms NEC	3 (0.4%)	5 (0.5%)	2 (0.3%)	10 (0.4%)
general signs and symptoms NEC	14 (2.0%)	21 (2.0%)	31 (4.7%)	66 (2.8%)
gingival disorders, signs, and symptoms NEC	1 (0.1%)	0 (0.0%)	1 (0.2%)	2 (0.1%)
headaches NEC	78 (11.2%)	105 (10.1%)	88 (13.3%)	271 (11.3%)
herpes viral infections	4 (0.6%)	4 (0.4%)	7 (1.1%)	15 (0.6%)
injection site reactions	1 (0.1%)	35 (3.4%)	2 (0.3%)	38 (1.6%)
joint related signs and symptoms	13 (1.9%)	17 (1.6%)	15 (2.3%)	45 (1.9%)
lacrimation disorders	1 (0.1%)	1 (0.1%)	0 (0.0%)	2 (0.1%)
lid, lash and lacrimal infections, irritations, and inflammations	1 (0.1%)	0 (0.0%)	0 (0.0%)	1 (0.0%)
lymphatic system disorders NEC	5 (0.7%)	10 (1.0%)	8 (1.2%)	23 (1.0%)
menstrual and uterine bleeding NEC	1 (0.1%)	2 (0.2%)	0 (0.0%)	3 (0.1%)
menstruation with increased bleeding	7 (1.0%)	3 (0.3%)	3 (0.5%)	13 (0.5%)
migraine headaches	1 (0.1%)	4 (0.4%)	6 (0.9%)	11 (0.5%)
mood alterations with depressive symptoms	3 (0.4%)	1 (0.1%)	2 (0.3%)	6 (0.3%)
muscle pains	7 (1.0%)	21 (2.0%)	12 (1.8%)	40 (1.7%)
muscle related signs and symptoms NEC	6 (0.9%)	2 (0.2%)	5 (0.8%)	13 (0.5%)
musculoskeletal and connective tissue conditions NEC	5 (0.7%)	4 (0.4%)	1 (0.2%)	10 (0.4%)
musculoskeletal and connective tissue pain and discomfort	107 (15.4%)	153 (14.7%)	32 (4.8%)	292 (12.2%)
nasal congestions and inflammation	1 (0.1%)	2 (0.2%)	2 (0.3%)	5 (0.2%)

nausea and vomiting symptoms	10 (1.4%)	25 (2.4%)	16 (2.4%)	51 (2.1%)
ocular disorders NEC	2 (0.3%)	0 (0.0%)	0 (0.0%)	2 (0.1%)
oedema NEC	1 (0.1%)	0 (0.0%)	0 (0.0%)	1 (0.0%)
oral dryness and saliva altered	3 (0.4%)	2 (0.2%)	2 (0.3%)	7 (0.3%)
osteoarthtropathies	2 (0.3%)	0 (0.0%)	0 (0.0%)	2 (0.1%)
pain and discomfort NEC	34 (4.9%)	65 (6.3%)	42 (6.4%)	141 (5.9%)
Papulosquamous conditions	1 (0.1%)	0 (0.0%)	0 (0.0%)	1 (0.0%)
paresthesia and dysesthesia	7 (1.0%)	2 (0.2%)	5 (0.8%)	14 (0.6%)
pericardial disorders NEC	1 (0.1%)	0 (0.0%)	0 (0.0%)	1 (0.0%)
physical examination procedures and organ system status	6 (0.9%)	10 (1.0%)	0 (0.0%)	16 (0.7%)
pneumothorax and pleural effusions NEC	1 (0.1%)	0 (0.0%)	0 (0.0%)	1 (0.0%)
pruritus NEC	2 (0.3%)	6 (0.6%)	2 (0.3%)	10 (0.4%)
rashes, eruptions and exanthemas NEC	3 (0.4%)	8 (0.8%)	7 (1.1%)	18 (0.8%)
skin and subcutaneous tissue viral infections	3 (0.4%)	2 (0.2%)	3 (0.5%)	8 (0.3%)
supraventricular arrhythmias	1 (0.1%)	0 (0.0%)	0 (0.0%)	1 (0.0%)
total fluid volume increase	1 (0.1%)	0 (0.0%)	0 (0.0%)	1 (0.0%)
upper respiratory signs and symptoms	2 (0.3%)	1 (0.1%)	2 (0.3%)	5 (0.2%)
upper respiratory tract infections NEC	7 (1.0%)	6 (0.6%)	7 (1.1%)	20 (0.8%)
upper respiratory tract signs and symptoms	9 (1.3%)	12 (1.2%)	16 (2.4%)	37 (1.5%)
urticaria	1 (0.1%)	0 (0.0%)	2 (0.3%)	3 (0.1%)
vaccination site reactions	113 (16.2%)	150 (14.4%)	52 (7.9%)	315 (13.1%)
vascular hypotensive disorders	2 (0.3%)	2 (0.2%)	0 (0.0%)	4 (0.2%)
vertigos NEC	13 (1.9%)	15 (1.4%)	16 (2.4%)	44 (1.8%)

viral upper respiratory tract infections	1 (0.1%)	5 (0.5%)	0 (0.0%)	6 (0.3%)
allergic conditions NEC	0 (0.0%)	1 (0.1%)	1 (0.2%)	2 (0.1%)
alopecias	0 (0.0%)	2 (0.2%)	0 (0.0%)	2 (0.1%)
angioedemas	0 (0.0%)	2 (0.2%)	0 (0.0%)	2 (0.1%)
bladder disorders NEC	0 (0.0%)	1 (0.1%)	1 (0.2%)	2 (0.1%)
breast signs and symptoms	0 (0.0%)	1 (0.1%)	0 (0.0%)	1 (0.0%)
cognitive and attention disorders and disturbances NEC	0 (0.0%)	3 (0.3%)	0 (0.0%)	3 (0.1%)
confusion and disorientation	0 (0.0%)	1 (0.1%)	0 (0.0%)	1 (0.0%)
dental pain and sensation disorders	0 (0.0%)	1 (0.1%)	0 (0.0%)	1 (0.0%)
depressive disorders	0 (0.0%)	1 (0.1%)	2 (0.3%)	3 (0.1%)
emotional and mood disturbances NEC	0 (0.0%)	1 (0.1%)	0 (0.0%)	1 (0.0%)
flatulence, bloating and distension	0 (0.0%)	1 (0.1%)	2 (0.3%)	3 (0.1%)
gingival Infections	0 (0.0%)	2 (0.2%)	0 (0.0%)	2 (0.1%)
heart rate and pulse investigations	0 (0.0%)	1 (0.1%)	1 (0.2%)	2 (0.1%)
lower respiratory tract signs and symptoms	0 (0.0%)	1 (0.1%)	0 (0.0%)	1 (0.0%)
muscle weakness conditions	0 (0.0%)	1 (0.1%)	0 (0.0%)	1 (0.0%)
myopathies	0 (0.0%)	1 (0.1%)	0 (0.0%)	1 (0.0%)
neurological signs and symptoms NEC	0 (0.0%)	4 (0.4%)	8 (1.2%)	12 (0.5%)
oral soft tissue signs and symptoms	0 (0.0%)	2 (0.2%)	0 (0.0%)	2 (0.1%)
pilar disorders NEC	0 (0.0%)	1 (0.1%)	0 (0.0%)	1 (0.0%)
pustular conditions	0 (0.0%)	1 (0.1%)	0 (0.0%)	1 (0.0%)
rate and rhythm disorders NEC	0 (0.0%)	2 (0.2%)	2 (0.3%)	4 (0.2%)
retinal structural change, deposit, and degeneration	0 (0.0%)	1 (0.1%)	0 (0.0%)	1 (0.0%)
sleep disorder NEC	0 (0.0%)	2 (0.2%)	0 (0.0%)	2 (0.1%)

tongue signs and symptoms	0 (0.0%)	1 (0.1%)	0 (0.0%)	1 (0.0%)
visual disorders NEC	0 (0.0%)	1 (0.1%)	0 (0.0%)	1 (0.0%)
visual impairment and blindness (excluding color blindness)	0 (0.0%)	1 (0.1%)	2 (0.3%)	3 (0.1%)
vulvovaginal disorders NEC	0 (0.0%)	1 (0.1%)	0 (0.0%)	1 (0.0%)
auditory nerve disorders	0 (0.0%)	0 (0.0%)	2 (0.3%)	2 (0.1%)
bladder and urethral symptoms	0 (0.0%)	0 (0.0%)	4 (0.6%)	4 (0.2%)
bladder infections and inflammations	0 (0.0%)	0 (0.0%)	1 (0.2%)	1 (0.0%)
conjunctival infections, irritations, and inflammations	0 (0.0%)	0 (0.0%)	1 (0.2%)	1 (0.0%)
haemorrhoids and gastrointestinal varices (excluding oesophageal)	0 (0.0%)	0 (0.0%)	1 (0.2%)	1 (0.0%)
meningitis NEC	0 (0.0%)	0 (0.0%)	1 (0.2%)	1 (0.0%)
nasal disorders NEC	0 (0.0%)	0 (0.0%)	7 (1.1%)	7 (0.3%)
ocular infections, inflammations, and associated manifestations	0 (0.0%)	0 (0.0%)	5 (0.8%)	5 (0.2%)
olfactory nerve disorders	0 (0.0%)	0 (0.0%)	1 (0.2%)	1 (0.0%)
sensory abnormalities NEC	0 (0.0%)	0 (0.0%)	1 (0.2%)	1 (0.0%)
somatic symptom disorders	0 (0.0%)	0 (0.0%)	1 (0.2%)	1 (0.0%)
tendon disorders	0 (0.0%)	0 (0.0%)	1 (0.2%)	1 (0.0%)
tremor (excl congenital)	0 (0.0%)	0 (0.0%)	1 (0.2%)	1 (0.0%)
vascular hypertensive disorders NEC	0 (0.0%)	0 (0.0%)	1 (0.2%)	1 (0.0%)

Supplementary Figure 2. Self-reported duration of adverse effects

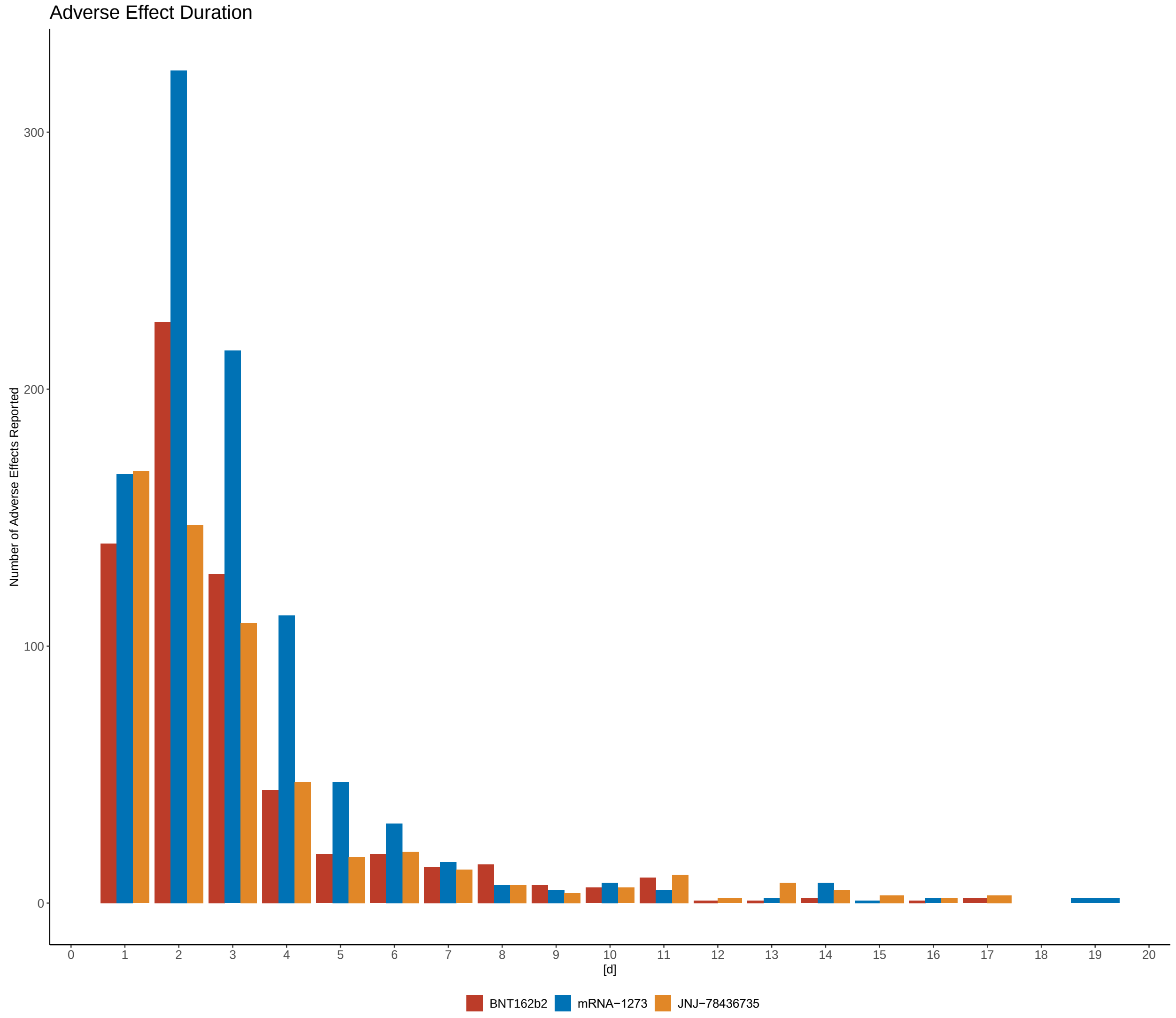

Supplementary Table 6. Self-reported severity of adverse effects by vaccine type

Severity	BNT162b2 (N=659)	mRNA-1273 (N=968)	JNJ-78436735 (N=606)	Overall (N=2233)
Very mild	119 (18.1%)	112 (11.6%)	58 (9.6%)	289 (13.0%)
Mild	271 (41.1%)	366 (37.8%)	170 (28.0%)	807 (36.1%)
Moderate	207 (31.4%)	334 (34.5%)	267 (44.1%)	808 (36.2%)
Severe	60 (9.1%)	144 (14.9%)	96 (15.8%)	300 (13.4%)
Very severe	2 (0.3%)	12 (1.2%)	15 (2.5%)	29 (1.3%)

Supplementary R Code. R code to reproduce findings and figures based on the submitted minimal dataset

```
####Setup####
library("readxl")
library("tidyverse")
library('gtsummary')
library("broom")
library("ggpubr")
library('fmsb')
library('hrbrthemes')
library('table1')
library('corrr')
library('RColorBrewer')
library('ggsci')
library('ggthemes')
library('xlsx')

#table1 and functions
render.cont <- function(x) { with(stats.default(x), c("", "Mean (SD)" = sprintf("%0.1f (%0.1f)", MEAN, SD), "Median
(IQR)" = sprintf("%0.1f (%0.1f to %0.1f)", MEDIAN, Q1, Q3), "Range" = sprintf("%0.0f to %0.0f", MIN, MAX))) }
##sets the settings for reporting of continuous variables (mean with SD and median with IQR and range- you can
change as you like)
render.cat <- function(x) { c("", sapply(stats.default(x), function(y) with(y, sprintf("%d (%0.1f%%)", FREQ,
PCTnoNA)))) } ##sets the settings for reporting of categorical variables (proportion and missing is not included in
the denominator)
`%!in%` <- Negate(`%in%`)

##import dataset
dataset  <- read_excel("AE_dataset.xlsx",
                       sheet = "data") %>%
  mutate(vaccine_type = factor(vaccine_type, levels = c("Pfizer/Biontech", "Moderna", "Johnson&Johnson")),
#recode factors
       vaccine_type_grp=recode_factor(vaccine_type_grp,
                       'mrna'='mRNA-Vaccines',
                    'vector'='JNJ-78436735'),
       vaccine_type=recode_factor(vaccine_type,
                  'Pfizer/Biontech'='BNT162b2',
                  'Moderna'='mRNA-1273',
```

```
                                'Johnson&Johnson'='JNJ-78436735'),
        app_use = factor(app_use, levels = c("Yes, always", "Yes, sometimes", "No"))) %>%
  mutate_at(vars(risk_homeoffice, risk_hygiene, risk_social_dist,risk_mask, risk_contact),
          funs(factor(., levels = c("Never/Impossible","Rarely","Sometimes","Frequently","Always")))) %>%
  mutate_at(vars(vacc_pharm, vacc_bag, vacc_immune, vacc_important, vacc_lifestyle),
          funs(factor(., levels = c("I disagree completely","Rather disagree","Neutral","Rather agree","I agree
completely"))))

####Table 1. Demographic and clinical characteristics of the study population####
dem_tbl <- dataset %>% #create dataframe with unique IDs
  group_by(record_id) %>%
  slice(1) %>%
  ungroup()

table1(~ age + age_grp + sex +
        preexisting_cond + hypertension + diabetes + cvd + respiratory + ckd + cancer + immune_supp + blood +
        smoking + education + ae_present + IgA_bl_stat + IgG_bl_stat + pcr_pos + pos_ab_pcr + ae_present|
vaccine_type,
      data = dem_tbl,
      render.continuous = render.cont,
      render.categorical = render.cat)


####Figure 2. Frequency of any, local and systemic adverse effects (five most common systemic and local adverse
effects)####
  ##Figure 2A: Overall adverse effects

dem_tbl %>%
    select(record_id, contains('local'), contains('systemic'), ae_present, - local_or_systemic) %>%
    mutate(record_id=ifelse(record_id>0,1,0)) %>%
  mutate_at(vars(any_local,local_pain,
local_erythema,local_lymphnodes,local_pruritus,local_swelling,any_systemic,systemic_pain,
              systemic_asthenia,systemic_chills,systemic_headache,systemic_pyrexia, ae_present),
          funs(case_when(. == "Yes" ~ 1,
                         . == "No" ~ 0,
                         TRUE ~ NA_real_))) %>%
    transmute_at(.vars = vars(record_id:ae_present),
              .funs = funs(sum(.,na.rm = T)/sum(record_id))) %>%
    pivot_longer(cols = -1,
              names_to = 'ae',
              values_to = 'Percentage') %>%
    distinct() %>%
    mutate(Percentage=Percentage*100,
        l_or_s=ifelse(ae=='ae_present','Any Adverse Effects',ifelse(ae=='any_local'|
                                                    ae=='local_erythema'|
                                                    ae=='local_swelling'|
                                                    ae=='local_pain'|
                                                    ae=='local_lymphnodes'|
                                                    ae=='local_pruritus','Local','Systemic')),
        ae=recode_factor(ae,
                         'ae_present'= 'Any Symptom ',
                         'any_systemic'= 'Any Systemic Symptom',
                         'any_local'='Any Local Symptom',
```

```
                        'local_pain'='Local Pain',
                        'local_erythema'='Local Erythema',
                        'local_swelling'='Local Swelling',
                        'local_lymphnodes'='Local Lymphnodes',
                        'local_pruritus'='Local Pruritus',
                        'systemic_pain'='Systemic Pain',
                        'systemic_asthenia'='Asthenia (Fatigue)',
                        'systemic_chills'='Chills',
                        'systemic_headache'='Headache',
                        'systemic_pyrexia'='Pyrexia'),
          Percentage=round(Percentage,digits = 2)) %>%
  arrange(desc(Percentage))%>%
  filter(ae!='record_id') %>%
  ggplot(aes(reorder(ae,-Percentage),Percentage, fill=l_or_s),)+
  geom_col(width = 0.3)+
  geom_text(aes(label = Percentage),vjust=-0.5, size=4)+
  geom_vline(xintercept = 1.5)+
  geom_vline(xintercept = 3.5)+
  theme_classic2()+
  theme(axis.text.x=element_text(angle = -90, hjust = 0, size = 14),
        axis.title.x = element_blank(),
        legend.title = element_blank(),
        axis.text.y = element_text(size = 14),
        legend.text = element_text(size = 14))+
  labs(title = "Overall",
       x='Adverse Effects',
       y="Percentage of Participants [%]",
       fill='Local or Systemic Adverse Effect')+
  theme(legend.position = 'bottom')+
  scale_fill_nejm()+
  scale_color_nejm()->gr_ae_overall


 ###Figure 2B:Percentage of adverse effects by vaccine
dem_tbl %>%
 select(record_id, contains('local'), contains('systemic'), ae_present, vaccine_type, - local_or_systemic) %>%
 mutate(record_id=ifelse(record_id>0,1,0)) %>%
 mutate_at(vars(any_local,local_pain,
local_erythema,local_lymphnodes,local_pruritus,local_swelling,any_systemic,systemic_pain,
              systemic_asthenia,systemic_chills,systemic_headache,systemic_pyrexia, ae_present),
          funs(case_when(. == "Yes" ~ 1,
                         . == "No" ~ 0,
                         TRUE ~ NA_real_))) %>%
 group_by(vaccine_type) %>%
 transmute_at(.vars = vars(record_id:ae_present),
             .funs = funs(sum(.,na.rm = T)/sum(record_id))) %>%
 pivot_longer(cols = -1,
             names_to = 'ae',
             values_to = 'Percentage') %>%
 distinct() %>%
 mutate(Percentage=Percentage*100,
       l_or_s=ifelse(ae=='ae_present','Any Adverse Effects',ifelse(ae=='any_local'|
                                    ae=='local_erythema'|
```

```
                                    ae=='local_swelling'|
                                    ae=='local_pain'|
                                    ae=='local_lymphnodes'|
                                    ae=='local_pruritus','Local','Systemic')),
         ae=recode_factor(ae,
                   'ae_present'= 'Any Symptom',
                   'any_systemic'= 'Any Systemic Symptom',
                   'any_local'='Any Local Symptom',
                   'local_pain'='Local Pain',
                   'local_erythema'='Local Erythema',
                   'local_swelling'='Local Swelling',
                   'local_lymphnodes'='Local Lymphnodes',
                   'local_pruritus'='Local Pruritus',
                   'systemic_pain'='Systemic Pain',
                   'systemic_asthenia'='Asthenia (Fatigue)',
                   'systemic_chills'='Chills',
                   'systemic_headache'='Headache',
                   'systemic_pyrexia'='Pyrexia'),
         checklist_vaccine1_typ=recode_factor(vaccine_type,
                                        'Pfizer/Biontech'='BNT162b2',
                                        'Moderna'='mRNA-1273',
                                        'Johnson&Johnson'='JNJ-78436735'),
         Percentage=round(Percentage,digits = 2)) %>%
  arrange(desc(Percentage))%>%
  filter(ae!='record_id') %>%
  ggplot(aes(reorder(ae,-Percentage),Percentage, fill=l_or_s),)+
  geom_col(width = 0.3)+
  geom_vline(xintercept = 1.5)+
  geom_vline(xintercept = 3.5)+
  theme_classic2()+
  geom_text(aes(label = Percentage),vjust=-0.5, size=4)+
  theme(axis.text.x=element_text(angle = -90, hjust = 0, size = 14),
        axis.title.x = element_blank(),
        axis.title.y = element_blank(),
        legend.title = element_blank(),
        axis.text.y = element_text(size = 14),
        legend.text = element_text(size = 14),
        strip.text = element_text(size=14))+
  labs(title = "By Vaccine",
       x='Adverse Effects',
       y="Percentage of Participants [%]",
       fill='Local or Systemic Adverse Effect')+
  facet_wrap(~checklist_vaccine1_typ, dir = 'v')+
  theme(legend.position = 'bottom')+
  scale_fill_nejm()+
  scale_color_nejm()->gr_ae_vacc

####Figure 3. Characteristics of self-reported adverse effects####
##Figure 3A: Adverse effect onset by vaccine type

dataset %>%
  select(-ae_treatment) %>%
  distinct() %>%
```

```
  drop_na(time_vaccine_ae,ae_SOC) %>%
  filter(time_vaccine_ae<40,time_vaccine_ae>0) %>%
  ggplot(aes(time_vaccine_ae,color=vaccine_type))+
  geom_freqpoly(binwidth=1)+
  scale_color_nejm()+
  theme_classic2()+
  theme(legend.position = 'bottom',
        axis.title.y = element_text(size = 12),
        axis.title.x = element_text(size = 12),
        axis.text = element_text(size = 12),
        title = element_text(size = 16),
        legend.text = element_text(size = 12),
        legend.title = element_blank())+
  scale_x_continuous(breaks = c(0:40))+
  labs(title = 'Onset',
       x='Time since Vaccination [d]',
       y='Number of Symptoms Reported',
       color='Vaccine Administered')->gr_onset

###Figure 3A Zoom View
dataset %>%
  select(-ae_treatment) %>%
  distinct() %>%
  drop_na(time_vaccine_ae,ae_SOC) %>%
  filter(time_vaccine_ae<40,time_vaccine_ae>0) %>%
  mutate(vaccine_type=recode_factor(vaccine_type,
                                    'Pfizer/Biontech'='BNT162b2',
                                    'Moderna'='mRNA-1273',
                                    'Johnson&Johnson'='JNJ-78436735'),) %>%
  ggplot(aes(time_vaccine_ae,color=vaccine_type))+
  geom_freqpoly(binwidth=1)+
  scale_color_nejm()+
  theme_classic2()+
  theme(legend.position = 'none',
        axis.title.y = element_blank(),
        axis.title.x = element_blank(),
        axis.text = element_text(size = 24),
        title = element_text(size=32),
        legend.text = element_text(size = 12),
        legend.title = element_blank())+
  scale_x_continuous(breaks = seq(0,40,2))+
  coord_cartesian(xlim = c(5, 40), ylim = c(0, 50), clip = "on")+
  labs(title = 'Zoom View Day 4 - 40',
       x='Time since Vaccination [d]',
       y='Number of Symptoms Reported',
       color='Vaccine Administered')

ggsave(plot = last_plot(), "graph2_zoom.pdf", height= 14, width= 20)
#insert this pdf in other graphics programm

###Figure 3B: Self-reported severity by vaccine type
dataset %>%
  select(-ae_treatment) %>%
```

```
  distinct() %>%
  drop_na(vaccine_type, ae_severity) %>%
  mutate(ae_severity  = factor(ae_severity, levels = c('Very Mild','Mild',
                                                       'Moderate','Severe',
                                                       'Very Severe'))) %>%
  ggplot(aes(fct_rev(vaccine_type) ,fill = ae_severity ))+
  geom_bar(position =position_fill(reverse = T) ,
           alpha = 1,
           width = 0.5)+
  coord_flip()+
  scale_y_continuous(labels=scales::percent)+
  theme_minimal()+
  scale_fill_brewer(palette = 'YlOrRd', direction = 1)+
  labs(title = 'Severity',
       y='% of All Reported Adverse Effects',
       x='',
       fill='Severity')+
  theme(legend.position = 'bottom',
        axis.title.y = element_text(size = 12),
        axis.title.x = element_text(size = 12),
        axis.text = element_text(size = 12),
        title = element_text(size = 16),
        legend.title = element_blank(),
        legend.text = element_text(size= 14))+
  guides(fill=guide_legend(nrow=2, byrow=TRUE))#->gr_sev


##Figure 3C: Consequences of adverse effects by vaccine

dataset %>%
  drop_na(ae_treatment) %>%
  select(ae_type, ae_severity, ae_treatment, vaccine_type) %>%
  mutate(ae_treatment=factor(ae_treatment, levels = c('Spontaneous Resolution',
                                      'Self-Medication',
                                      'Remote Consultation/ Pharmacy',
                                      'ER or Physician Visit',
                                      'Hospitalization'))) %>%
  ggplot(aes(fct_rev(vaccine_type), fill=ae_treatment))+
  geom_bar(position = position_fill(reverse = T),
           width = 0.5)+
  coord_flip()+
  scale_y_continuous(labels=scales::percent)+
  theme_minimal()+
  scale_fill_brewer(palette = 'YlOrRd', direction = 1 )+
  labs(x='Administered Vaccine',
       y='% of All Reported Adverse Effects',
       title = 'Consequences',
       fill='Consequence')+
  theme(legend.position = 'bottom',
        axis.title.y = element_blank(),
        axis.ticks.y = element_blank(),
        axis.text.y = element_blank(),
        title = element_text(size = 16),
```

```
      axis.title.x = element_text(size = 12),
      legend.title = element_blank(),
      legend.text = element_text(size= 14))+
  guides(fill=guide_legend(nrow=2, byrow=TRUE))#->gr_reac


####Figure 4####
##Figure 4A: Trust in vaccine and institutions
dem_tbl %>%
  select(vaccine_type_grp, vacc_pharm, vacc_bag, vacc_immune, vacc_important, vacc_lifestyle) %>%
  pivot_longer(cols = -1,names_to = 'question',values_to = 'opinion' ) %>%
  mutate( question=recode_factor(question,
                              'vacc_bag'='I trust the Federal Office of Public Health (FOPH).',
                              'vacc_pharm'='I trust the vaccine producers and pharmaceutical companies in general.',
                              'vacc_immune'='I understand how vaccines help my body to fend off infectious disease.',
                              'vacc_lifestyle'='Vaccines are part of a healthy lifestyle.',
                              'vacc_important'='I think it is important to be vaccinated.'
                              )) %>%
  drop_na(opinion) %>%
  ggplot(aes(question, fill=opinion))+
  geom_bar(position = position_fill(reverse = T),
         width = 0.3)+
  coord_flip()+
  facet_wrap(~ vaccine_type_grp, dir = 'h')+
  scale_y_continuous(labels=scales::percent)+
  theme_minimal()+
  scale_fill_brewer(palette = 'PRGn' )+
  theme(legend.position = 'bottom',
      axis.title.y = element_blank(),
      legend.title = element_blank(),
      axis.text.y = element_text(size = 14),
      legend.text = element_text(size = 12),
      title = element_text(size = 16, face = 'bold'),
      strip.text = element_text(size = 14,  face = 'bold', hjust = 0),
      plot.margin = margin(1,1,80,1))+
  labs(title = 'Trust in Vaccine and Institutions',
     x='Statement',
     y='')#->gr_trust



  ##Figure 4B: Compliance with Recommended Public Health Measures
dem_tbl %>%
  select(vaccine_type_grp,contains('risk')) %>%
  pivot_longer(cols = -1,names_to = 'question',values_to = 'opinion' ) %>%
  mutate(question=recode_factor(question,
                              'risk_homeoffice'='I was able to work from home.',
                              'risk_hygiene'='I was able to adhere to recommended hygiene guidelines.',
                              'risk_social_dist'='I was able to practice social distancing.',
                              'risk_mask'='I wore a mask when away from home.',
                              'risk_contact'='I avoided larger crowds.')) %>%
  drop_na(opinion) %>%
  ggplot(aes(question, fill=opinion))+
```

```
  geom_bar(position = position_fill(reverse = T),
           width = 0.3)+
  coord_flip()+
  facet_wrap(~vaccine_type_grp, dir = 'h')+
  scale_y_continuous(labels=scales::percent)+
  theme_minimal()+
  scale_fill_brewer(palette = 'PRGn' )+
  theme(legend.position = 'bottom',
        axis.title.y = element_blank(),
        legend.title = element_blank(),
        axis.text.y = element_text(size = 14),
        legend.text = element_text(size = 12),
        title = element_text(size = 16, face = 'bold'),
        strip.text = element_blank(),
        plot.margin = margin(1,1,80,1))+
  labs(title = 'Compliance with Recommended Public Health Measures',
       x='Statement',
       y='')#->gr_risk


  ## Figure 4C: Swiss Covid App Use
dem_tbl %>%
  select(vaccine_type_grp,app_use) %>%
  pivot_longer(cols = -1,names_to = 'question',values_to = 'opinion' ) %>%
        mutate(question=recode_factor(question,
                                'app_use'='Do you currently use the Swiss Covid App?'))%>%
  drop_na(opinion) %>%
  ggplot(aes(question, fill=opinion))+
  geom_bar(position = position_fill(reverse = F),
           width = 0.3)+
  coord_flip()+
  facet_wrap(~vaccine_type_grp, dir = 'h')+
  scale_y_continuous(labels=scales::percent)+
  theme_minimal()+
  scale_fill_brewer(palette = 'PRGn', direction = -1,
                    start)+
  theme(legend.position = 'bottom',
        axis.title.y = element_blank(),
        legend.title = element_blank(),
        axis.text.y = element_text(size = 14),
        legend.text = element_text(size = 12),
        title = element_text(size = 16, face = 'bold'),
        strip.text = element_blank())+
  labs(title = 'Swiss Covid App Use',
       x='Statement',
       y='')#->gr_app_use



####Creating Final Graphs####

#Prevalence
ggarrange(gr_ae_overall,gr_ae_vacc,
```

```
          labels = c('A','B'),
          ncol = 2,
          align = 'hv',
          common.legend = T,
          legend = 'bottom')->gr_1

gr_1 <- annotate_figure(gr_1,
               top = text_grob('Prevalence of Adverse Effects',
                               face = 'bold',
                               size = 14
               ))

ggsave(plot = last_plot(), "Figure-2.eps", height= 17, width= 16)

#Symptom Characteristics
ggarrange(gr_onset,
          labels = 'A',
          font.label = list(size=16),
          ncol = 1,
          align = 'hv',
          common.legend = T,
          legend = 'bottom')->gr_dynamics

ggarrange(gr_sev,gr_reac,
          labels = c('B','C'),
          font.label = list(size=16),
          nrow = 1,
          align = 'hv')->gr_sevreac

ggarrange(gr_dynamics,gr_sevreac,
          ncol = 1,
          heights = c(5,1.5),
          align = 'hv')->gr_2

gr_2<-annotate_figure(gr_2,
               top = text_grob('Symptom Characteristics by Vaccine',
                               face = 'bold',
                               size = 16
                               ))

ggsave(plot = last_plot(), "graph2.pdf", height= 14, width= 20)

ggsave(plot = last_plot(), "Figure-3.eps", device = "eps", height= 14, width= 20)


#Trust
gr_3<-ggarrange(gr_trust,gr_risk,gr_app_use,
          labels = c('A','B','C'),
          font.label = list(size=16),
          heights = c(5,5,3.2),
          ncol = 1,
          align = 'hv')
```

```
ggsave(plot = last_plot(), "Figure-4.tiff", device = "tiff", height= 14, width= 16)

ggsave(plot = last_plot(), "Figure-4.eps", device = "eps", height= 14, width= 16)



# Supplementary Material -------------------------------------------------
##Supplementary Figure 1: Answers to trust related questions among mRNA vaccine recipients
dem_tbl %>%
  filter(vaccine_type!='JNJ-78436735') %>%
  select(vaccine_type,vacc_pharm,vacc_bag,vacc_immune,vacc_important,vacc_lifestyle) %>%
  pivot_longer(cols = -1,names_to = 'question',values_to = 'opinion' ) %>%
  mutate(opinion=recode_factor(opinion,
                               '1'='I disagree completely',
                               '2'='Rather disagree',
                               '3'='Neutral',
                               '4'='Rather agree',
                               '5'='I agree completely'),
         question=recode_factor(question,
                                'vacc_bag'='I trust the Federal Office of Public Health (FOPH).',
                                'vacc_pharm'='I trust the vaccine producers and pharmaceutical companies in general.',
                                'vacc_immune'='I understand how vaccines help my body to fend off infectious disease.',
                                'vacc_lifestyle'='Vaccines are part of a healthy lifestyle.',
                                'vacc_important'='I think it is important to be vaccinated.'
         )) %>%
  drop_na(opinion) %>%
  ggplot(aes(question, fill=opinion))+
  geom_bar(position = position_fill(reverse = T),
           width = 0.3)+
  coord_flip()+
  facet_wrap(~vaccine_type, dir = 'v')+
  scale_y_continuous(labels=scales::percent)+
  theme_minimal()+
  scale_fill_brewer(palette = 'PRGn' )+
  theme(legend.position = 'bottom',
        axis.title.y = element_blank(),
        legend.title = element_blank(),
        axis.text.y = element_text(size = 16),
        legend.text = element_text(size = 14))+
  labs(title = 'Trust in Vaccine and Institutions',
       x='Statement',
       y='[%]')

ggsave(plot = last_plot(), "Supplement_mRNA_trust.pdf", height= 14, width= 16)


####Supplementary Table 2. Demographic and clinical characteristics of participants, with BNT162b2 and mRNA-
1273 combined into mRNA vaccine group ####
dem_tbl %>%
table1(~ age + age_grp + sex +
```

```
         preexisting_cond + hypertension + diabetes + cvd + respiratory + ckd + cancer + immune_supp + blood +
         smoking + education + ae_present + IgA_bl_stat + IgG_bl_stat + pcr_pos + pos_ab_pcr + ae_present|
vaccine_type_grp,
      render.continuous = render.cont,
      render.categorical = render.cat,
      data=.)


####Supplementary Table 3. Factors associated with adverse effect development after SARS-CoV2 vaccination
####
dem_tbl <- dem_tbl %>%
  mutate(ae_present=factor(ae_present, levels = c("No", "Yes")),
         age_grp  = factor(age_grp, levels = c('≥65','<65')),
         sex= factor(sex, levels = c('male','female')),
         education = recode_factor(education,
                          'None or mandatory school'='None or mandatory school',
                          'Vocational training or specialized baccalaureate'='Vocational training or specialized
baccalaureate',
                          'Higher technical school or college'='Higher Education',
                          'University'='Higher Education'),
         smoking=recode_factor(smoking,
                        'daily'='smoker',
                        'occasionally'='smoker',
                        'former smoker'='former smoker',
                        'non smoker'='non smoker'),
         smoking=recode_factor(smoking, ref = 'non-smoker'))


##final model and regression table
library(broom)
m1 <-  glm(ae_present~age_grp+sex+ bmi+vaccine_type + education ,
          family = 'binomial' ,
          data = dem_tbl )

regression_table <- bind_rows (
  tidy(glm(ae_present~age_grp+sex+bmi + vaccine_type + education ,
          family = 'binomial' ,
          data = dem_tbl ), conf.int = T)[-1,],
  tidy(glm(ae_present~preexisting_cond + age_grp+sex+bmi+vaccine_type + education,
          family = 'binomial' ,
          data = dem_tbl ), conf.int = T)[2,],
  tidy(glm(ae_present~trust_vacc + age_grp+sex+bmi+vaccine_type + education,
          family = 'binomial' ,
          data = dem_tbl ), conf.int = T)[2,],
  tidy(glm(ae_present~pos_ab_pcr + age_grp+sex+bmi+vaccine_type + education,
          family = 'binomial' ,
          data = dem_tbl ), conf.int = T)[2,],
  tidy(glm(ae_present~smoking + age_grp+sex+bmi+vaccine_type + education,
           family = 'binomial' ,
           data = dem_tbl ), conf.int = T)[2:3,]
) %>%
  mutate(OR = exp(estimate), ci.lb = exp(conf.low), ci.ub= exp(conf.high),
         CI = paste(format(OR, format = "f", digits = 2), " (",format(ci.lb,
```

```
                                                  format = "f", digits =2)," to ", format(ci.ub, format="f", digits = 3), ")" , sep =
""),
 )  %>%
 select(term, OR, ci.lb, ci.ub, CI, p.value)


## Supplementary Table 4. Shift from local to systemic adverse effects after 2nd dose
dataset %>%
 select(-ae_treatment) %>%
 distinct() %>%
 drop_na(local_or_systemic) %>%
 filter(vaccine_type=='BNT162b2') %>% #enter any of the three vaccine types
 table1(~ local_or_systemic + vaccine_type|ae_vacc_dose , render.continuous = render.cont, render.categorical =
render.cat, data = .)


## Supplementary Table 5. Frequency of adverse effects according to MedDRA high level terms in alphabetic order

dataset %>%
 select(-ae_treatment) %>%
 distinct() %>%
 drop_na(ae_HLT) %>%
 table1(~ ae_HLT|vaccine_type , render.continuous = render.cont, render.categorical = render.cat, data = .)

##Supplementary Figure 2. Self-reported duration of adverse effects

dataset %>%
 select(-ae_treatment) %>%
 distinct() %>%
 drop_na(vaccine_type) %>%
 filter(ae_duration>0,ae_duration<20) %>%
 ggplot(aes(ae_duration,fill=vaccine_type))+
 geom_bar(position = 'dodge')+
 scale_fill_nejm()+
 theme_classic2()+
 theme(legend.position = 'bottom')+
 scale_x_continuous(breaks = c(0:20))+
 labs(title = 'Duration',
     x='Duration [d]',
     y='Number of Symptoms Reported',
     fill='Vaccine Administered')


 ## Supplementary Table 6. Self-reported severity of adverse effects
 dataset %>%
   select(-ae_treatment) %>%
   distinct() %>%
   drop_na(ae_severity) %>%
   mutate(ae_severity  = factor(ae_severity, levels = c('Very Mild','Mild',
                                          'Moderate','Severe',
                                           'Very Severe'))) %>%
   table1(~ ae_severity  | vaccine_type,
          render.continuous = render.cont, render.categorical = render.cat,
```

data = .)
