# Supplementary material for: Adverse effects, perceptions and attitudes related to BNT162b2, mRNA-1273 or JNJ-78436735 SARS-CoV-2 vaccines: Population-based cohort
Source: NPJ Vaccines. 2023 Apr 24;8:61. doi: 10.1038/s41541-023-00657-3 (PMC10123463; doi:10.1038/s41541-023-00657-3)
Supplement: Supplementary file 3 — REPORTING SUMMARY [file 41541_2023_657_MOESM3_ESM.pdf]

nature portfolio

Corresponding author(s): Anja Frei, PhD; anja.frei@uzh.ch

Last updated by author(s): Oct 7, 2022

Reporting Summary

Nature Portfolio wishes to improve the reproducibility of the work that we publish. This form provides structure for consistency and transparency in reporting. For further information on Nature Portfolio policies, see our Editorial Policies and the Editorial Policy Checklist.

Statistics

For all statistical analyses, confirm that the following items are present in the figure legend, table legend, main text, or Methods section.

n/a	Confirmed	
☐	☒	The exact sample size (*n*) for each experimental group/condition, given as a discrete number and unit of measurement
☒	☐	A statement on whether measurements were taken from distinct samples or whether the same sample was measured repeatedly
☐	☒	The statistical test(s) used AND whether they are one- or two-sided<br>*Only common tests should be described solely by name; describe more complex techniques in the Methods section.*
☐	☒	A description of all covariates tested
☐	☒	A description of any assumptions or corrections, such as tests of normality and adjustment for multiple comparisons
☐	☒	A full description of the statistical parameters including central tendency (e.g. means) or other basic estimates (e.g. regression coefficient) AND variation (e.g. standard deviation) or associated estimates of uncertainty (e.g. confidence intervals)
☐	☒	For null hypothesis testing, the test statistic (e.g. *F*, *t*, *r*) with confidence intervals, effect sizes, degrees of freedom and *P* value noted<br>*Give P values as exact values whenever suitable.*
☒	☐	For Bayesian analysis, information on the choice of priors and Markov chain Monte Carlo settings
☒	☐	For hierarchical and complex designs, identification of the appropriate level for tests and full reporting of outcomes
☐	☒	Estimates of effect sizes (e.g. Cohen's *d*, Pearson's *r*), indicating how they were calculated

Our web collection on statistics for biologists contains articles on many of the points above.

Software and code

Policy information about availability of computer code

Data collection	Immune assay data:<br>Luminex analysis of Spike-specific IgA and IgG, Nucleocapsid-specific IgG and SARS-CoV-2 Spike neutralization activity analysis: Samples were read on a Bio-Plex (Luminex) 200 plate reader with Bio-Plex Manager software (version 6.2; Bio-Rad).<br>Elecsys analysis of Spike-Specific and Nucleocapsid-Specific Ig: Sample were analyzed using a Cobas e411 analyzer instrument (software version 03.02; Roche).<br><br>Electronic survey data:<br>The Research Electronic Data Capture (REDCap) platform was used for data collection (current version 12.5.9) for all electronic survey data. REDCap is a publicly available, web-based application created and distributed by Vanderbilt University.
Data analysis	All analyses were performed using R (version 4.1.2).

For manuscripts utilizing custom algorithms or software that are central to the research but not yet described in published literature, software must be made available to editors and reviewers. We strongly encourage code deposition in a community repository (e.g. GitHub). See the Nature Portfolio guidelines for submitting code & software for further information.

Data

Policy information about availability of data

All manuscripts must include a data availability statement. This statement should provide the following information, where applicable:

- Accession codes, unique identifiers, or web links for publicly available datasets
- A description of any restrictions on data availability
- For clinical datasets or third party data, please ensure that the statement adheres to our policy

A minimal, deidentified dataset has been made available as a supplement to this publication together with the respective R code to reproduce all figures, tables and findings of this publication.

Human research participants

Policy information about studies involving human research participants and Sex and Gender in Research.

Reporting on sex and gender	In this study, we collected data on participants' self-reported sex. Data was collected in German, using standard terminology. We present sex distribution in our study.
Population characteristics	The study population characteristics are reported in detail in Table 1. In the overall study population of 575 individuals seeking basic immunization with BNT162b2, mRNA-1273 or JNJ-78436735 SARS-CoV-2 vaccines, population characteristics were as follows: Age: median 59 (IQR 41 to 70); Female: 323/575 (56.2%); number of BNT162b2 207/575 (36%) ,number of mRNA-1273 203/575 (35%), number of JNJ-78436735 165/575 (29%), preexisting conditions in 42%
Recruitment	We recruited participants between March 10, 2021, and January 27, 2022, at the University of Zurich's (UZH) vaccination center, the reference center for the Canton of Zurich, Switzerland. All individuals scheduled to receive one of the SARS-CoV-2 vaccines approved in Switzerland in 2021, BNT162b2 (Pfizer-BioNTech), mRNA1273 (Moderna), or JNJ-78436735 (Johnson & Johnson), were screened for eligibility. Eligibility criteria were being 18 years or older, being able to follow study procedures, having sufficient knowledge of the German language and residing in the Canton of Zurich.
Ethics oversight	The ethics committee of the Canton of Zurich (BASEC 2021-00273).

Note that full information on the approval of the study protocol must also be provided in the manuscript.

Field-specific reporting

Please select the one below that is the best fit for your research. If you are not sure, read the appropriate sections before making your selection.

☒ Life sciences ☐ Behavioural & social sciences ☐ Ecological, evolutionary & environmental sciences

For a reference copy of the document with all sections, see nature.com/documents/nr-reporting-summary-flat.pdf

Life sciences study design

All studies must disclose on these points even when the disclosure is negative.

Sample size	We included 575 randomly selected vaccinated individuals. Sample sizes were predetermined prior to initiation of the study and determined according to the primary objective of assessing immune responses. We assumed that the calculated sample size will be enough to capture the variability and range of immune responses that can be seen in vaccinated individuals. Based on our experiences and findings from other studies, we determined that 100 individuals per stratum of interest (age and vaccine type) would be sufficient. We included 207 participants for BNT162b2 and 203 for mRNA1273. However, we could not reach the predetermined sample size for JNJ-78436735 due to insufficient demand in the population. For JNJ-78436735 we included 165 participants.
Data exclusions	We excluded individuals who had already received a first dose of a SARS-CoV-2 vaccine. A daily age-stratified (18-64 years, 65 years or older) random sample was selected separately for each approved vaccine from all eligible individuals belonging to the following vaccination groups as defined by the Canton of Zurich 16,17: "Over 75 years", "over 65 years", "between 50–64 years", and "between 18–49 years". We excluded individuals belonging to groups specific for "healthcare workers", "caretakers of high-risk patients", "individuals living in communal facilities", and "individuals with the highest risk diseases" to ensure that that our sample was representative of the general population.<br>We excluded missing data and all reported adverse effects starting within three days before and at any timepoint after positive SARS-CoV-2 tests to ensure that reported symptoms were related to vaccination rather than infection.
Replication	no experiments were done due to the observational nature of this study.
Randomization	No randomization was performed due to the observational nature of this study.
Blinding	No group allocation was performed due to the observational nature of this study.

Reporting for specific materials, systems and methods

We require information from authors about some types of materials, experimental systems and methods used in many studies. Here, indicate whether each material, system or method listed is relevant to your study. If you are not sure if a list item applies to your research, read the appropriate section before selecting a response.

Materials & experimental systems

n/a	Involved in the study
☒	☐ Antibodies
☒	☐ Eukaryotic cell lines
☒	☐ Palaeontology and archaeology
☒	☐ Animals and other organisms
☐	☒ Clinical data
☒	☐ Dual use research of concern

Methods

n/a	Involved in the study
☒	☐ ChIP-seq
☒	☐ Flow cytometry
☒	☐ MRI-based neuroimaging

Clinical data

Policy information about clinical studies

All manuscripts should comply with the ICMJE guidelines for publication of clinical research and a completed CONSORT checklist must be included with all submissions.

Clinical trial registration	no clinical trial
Study protocol	The study protocol was prospectively registered on the International Standard Randomized Controlled Trial Number Registry (ISRCTN 15499304).
Data collection	We recruited participants between March 10, 2021, and January 27, 2022, at the University of Zurich's (UZH) vaccination center, the reference center for the Canton of Zurich, Switzerland. All individuals scheduled to receive one of the SARS-CoV-2 vaccines approved in Switzerland in 2021, BNT162b2 (Pfizer-BioNTech), mRNA1273 (Moderna), or JNJ-78436735 (Johnson & Johnson), were screened for eligibility.
Outcomes	Our primary outcomes included period prevalence, onset, duration, and severity of self-reported adverse effects over 12 weeks following vaccination, with a specific focus on the proportion of participants reporting allergic reactions, menstrual irregularities, or cardiac adverse effects, or requiring hospitalization. Secondary outcomes included risk factors associated with adverse effect reports, general perceptions and attitudes regarding vaccination, trust in public health authorities and pharmaceutical companies, and compliance with recommended public health measures.
